# Emergent poverty traps at multiple levels impede social mobility

**DOI:** 10.1057/s41599-025-06089-9

**Published:** 2025-11-19

**Authors:** Charles Dupont, Debraj Roy

**Affiliations:** 1 University of Amsterdam, Amsterdam, Netherlands; 2 Univeristy of Amsterdam, Amsterdam, Netherlands

**Keywords:** Economics, Development studies

## Abstract

Eradicating extreme poverty and inequality are the key leverage points to achieve the seventeen Sustainable Development Goals (SDGs). However, the reduction of extreme poverty and inequality remains vulnerable to shocks such as pandemics and climate change. Numerous models have been developed to examine the complex social interactions giving rise to inequality and persistent poverty, yet few approaches include multilevel dynamics. Here, we introduce a heterogeneous agent-based model to identify conditions underlying poverty traps at different levels, which manifest as distinct statistical steady-state outcomes. We find that vulnerabilities emerge from the interaction between individual and institutional mechanisms. Individual characteristics like risk aversion, attention, and saving propensity can lead to sub-optimal diversification and low capital accumulation. These individual drivers are reinforced by institutional mechanisms such as lack of financial inclusion, access to technology, and economic segregation, leading to persistent inequality and poverty traps. Our experiments demonstrate that addressing the above factors yields a “double dividend”—reducing poverty and inequality within and between communities and creating positive feedback that can withstand shocks. Finally, we demonstrate that our theoretical model can be used as a sandbox for cost-benefit analysis of intervention strategies.

## Introduction

At the forefront of the Sustainable Development Goals lies one of the most important and unresolved global challenges of our time: eradicating extreme poverty and reducing inequality within and among countries. Although poverty rates have decreased over the years, nearly 10% of the world’s population still lives in extreme poverty, characterized by a lack of basic needs such as access to food, clean drinking water, education, and information (Hasell et al., 2022). Emerging yet inconclusive evidence suggests that COVID-19 may have been a factor in this positive trend (Chancel et al., 2021; Yonzan et al., 2023). Estimates for 2023 indicate that poverty rates have likely returned to 2019 levels, with about 691 million people (8.6% of the world population) living in extreme poverty (Yonzan et al., 2023). The pandemic has also caused the largest increase in inequality between countries in three decades, and reducing inequality within and between countries is a scientific and a policy challenge (Chancel et al., 2021). In addition, climate change is proving to be a major obstacle for impoverished households and communities to adapt and develop in a sustainable way (Chancel et al., 2021). A recent review by Méjean et al. has indicated that the poor are expected to be disproportionately affected by the increasing frequency and intensity of climate-related shocks, potentially reversing progress towards poverty reduction and further increasing economic inequality (Méjean et al., 2024).

High levels of economic inequality can exacerbate poverty by concentrating wealth and opportunities among a small segment of the population, leaving others with limited resources and prospects for advancement. Poverty can act as a barrier to economic mobility—understood here as the ability of individuals and communities to improve their economic standing and escape low-income circumstances over time, rather than strictly as rank-order changes within a fixed distribution—as individuals born into poverty often face systemic obstacles such as inadequate access to education, healthcare, and employment opportunities. Limited economic mobility can perpetuate cycles of poverty, where individuals and possibly entire communities remain trapped in low-income circumstances across generations. Reflecting on the trajectory of economic development reveals a transition from a state characterized by extreme poverty to one marked by pervasive inequality. The empirical evidence highlights two key facts.

### Multi-level poverty traps

The alleviation of extreme poverty has progressed at a slow pace in Sub-Saharan Africa, and trends indicate an increase in poverty levels within the regions of the Middle East and North Africa, suggesting the presence of “poverty traps” (see Appendix A for a brief review). This trap involves self-reinforcing mechanisms that keep regions, countries, and neighbourhoods in a cycle of poverty (Azariadis and Stachurski, 2005). Barrett et al. (Barrett and Carter, 2013; Barrett and Swallow, 2006; Barrett et al., 2016; Santos and Barrett, 2018) and Adato et al. (Adato et al., 2013) provide evidence of multiple equilibrium in asset dynamics in rural Kenya, Madagascar, and South Africa. Evidence from rural China (Jalan and Ravallion, 2002) and Bangladesh (Balboni et al., 2022) shows that living in poor areas diminishes the productivity of a farmer’s investments, with inadequate community capital, such as limited infrastructure, potentially trapping households in poverty. An empirical survey (Kraay and McKenzie, 2014) examined mechanisms like S-shaped savings functions, coordination failures, hunger-based traps, and occupational poverty traps. The survey suggests that such traps are rare and mostly confined to remote or disadvantaged areas (Kraay and McKenzie, 2014). Emerging research highlights behavioural and geographic poverty traps as more plausible (Kraay and McKenzie, 2014). Recent research has observed that poverty traps often operate across multiple levels, prompting the conceptualization of multi-level or fractal poverty traps (Barrett and Swallow, 2006). The implication of the poverty trap at multiple levels for policy is significant, suggesting that cross-level interactions can reinforce or mitigate poverty traps (Barrett et al., 2018; Radosavljevic et al., 2021).

### Persistent horizontal inequality

Inequality appears at various spatial scales, from global to local, affecting regions, countries, cities, and neighbourhoods differently. In recent decades, while global inequalities between countries have decreased, inequalities within countries have surged. For example, China’s sudden shift from extreme poverty to extreme inequality is far from being an exception, as many other countries today face similar challenges (World Bank and the Development Research Center of the State Council, The People’s Republic of China, 2022; Qureshi, 2023; Xie and Zhou, 2014). Within cities, inequality can be stark between different neighbourhoods (e.g., formal vs. informal (Mutlu et al., 2023, Roy et al., 2018b)) and group identities (e.g., ethnicity, religion (Roy et al., 2018a)). There are many persistent horizontal inequalities in developing countries, such as northern groups in Nigeria or Ghana, Somalis in Kenya, Hutus in Rwanda and Burundi, or Muslims in India. Inequality can stem from differences in education (Galor and Zeira, 1993, Stiglitz, 1973), access to resources (Goderis and Malone, 2011) and technology (Aghion et al., 1999), individual risk aversion (Aghion and Bolton, 1992, Banerjee and Newman, 1991, Mengesha and Roy, 2025) and saving propensity (Banerjee and Yakovenko, 2010, Chakraborti and Chakrabarti, 2000), globalization and government policies (Chong and Gradstein, 2007). In conclusion, poverty traps and inequality are deeply intertwined, with high inequality often contributing to the persistence of poverty traps.

Although there is clear evidence of poverty traps and persistent inequality at multiple levels, the impact of cross-level interactions in poverty and emerging inequality is not well understood. The concept of multiple equilibria in current poverty traps literature typically describes scenarios where different initial conditions lead to divergent outcomes. However, this contrasts with interpretations in multi-agent game-theoretic models, where identical initial conditions can produce multiple distinct outcomes due to factors such as strategic complementarities, coordination failures, and macroeconomic fluctuations. Furthermore, current models attribute income variation primarily to differences in preferences and endowments, neglecting the role of variations in risky outcomes. This approach is logically incomplete, as it fails to explore what the long-term wealth distribution would look like in a scenario where agents start with identical endowments and preferences.

While a number of agent-based models have examined inequality and poverty, our understanding of the co-evolution of poverty traps across individual and community levels, driven by behavioural heterogeneity, remains a key challenge. Fierro et al. have investigated how monetary policy is impacted by income inequality using a macroscopic agent-based model (Fierro et al., 2023). Caiani et al. analysed the relationship between inequality and economic development with an agent-based stock-flow consistent model with different worker classes (Caiani et al., 2019). Botta et al. examined interactions between finance, instability, and inequality, also using an agent-based stock-flow consistent macroeconomic model (Botta et al., 2021). Palagi et al. studied interactions between inequality, income dynamics, and credit constraints in a generational agent-based model of households (Palagi et al., 2023). Although valuable in illuminating different facets of poverty and inequality, these approaches do not explicitly model emergent multi-level dynamics of poverty traps arising from interactions across different scales.

In response to these challenges, we have developed a two-level agent-based model that is novel in its study of the linkages between heterogeneous individual behaviour, community structure, and differential institutional access on emergent inequality and poverty at both individual and community levels. Individual agents are connected in a social network from which we extract communities. We construct an economy where heterogeneous agents experience uninsurable idiosyncratic shocks and smooth consumption. Agents are risk-averse and have identical preferences deriving utility from consumption and can engage in two types of productive projects: one safe but low-return project and several high-risk, high-return projects. Agents within the same community can form self-financing groups and undertake joint risk-sharing investment projects. Agents have an attention mechanism that allows agents to periodically update their investment portfolios according to observed realisations of project returns (see “Methods” section for details). In order to study the model’s behaviour, we construct Monte Carlo (Sobol) samples and run several repetitions for each combination of parameters in order to capture stochastic effects (Saltelli, 2002) (see “Experiments” in “Methods” for details). Then, we categorise the model runs at both the micro (agent) and meso (community) levels according to the evolution of wealth trajectories, which tend towards characteristic long-run steady states for the given parameter sets. We find robust evidence with all experimental setups for the existence of three distinct regimes: a persistent poverty trap for all (“All Poor”), a state of high inequality where some escape poverty (“Some Rich”), and a rarer scenario where all prosper (“All Rich”). The application of a manifold learning-based GSA method (see Appendix B) allows us to identify critical parameter regions responsible for giving rise to each regime, and we observe important differences in the sensitivity indices of these parameters at different temporal scales as well as micro (agent), meso (community), and macro (population) scales. Intervention experiments indicate that it is not possible to extricate agents from poverty in the All Poor regime, while interventions of sufficient magnitude can help to alleviate poverty to some degree for the Some Rich regime. We find key individual (behavioural) and community (institutional) mechanisms that are important to achieve sustainable reduction in poverty and inequality. At the individual level, behavioural characteristics like risk aversion, attention, and saving propensity can lead to sub-optimal diversification and low capital accumulation. At the community level, institutional drivers such as lack of financial inclusion, access to technology, and economic segregation are key drivers of inequality and poverty traps. Our results show that targeting horizontal inequality (between-community inequality) increases the rate of successful interventions through feedback.

## Methods

In this section, we describe the key components of the agent-based model as well as relevant theory.

### Agent social network

Homophily, the tendency for similar agents to form social connections, has been shown to play a significant role in how social networks are organised. Indeed, most people’s social networks are relatively homogeneous with respect to socioeconomic and demographic characteristics such as race and ethnicity, age, education, and household income (McPherson et al., 2001). This has significant implications for the kind of information that people have access to, as well as the behaviours that we tend to adopt. In a recent paper, Talaga and Nowak propose a Social Distance Attachment (SDA) model for randomly constructing social networks based on homophily (Talaga and Nowak, 2020). Furthermore, they show that their model is able to reproduce many of the characteristic features of social networks, making it an appealing choice for our present purposes. Existing poverty trap literature (Chantarat and Barrett, 2012) highlights the complex relationship between social networks, wealth distribution, and poverty alleviation, emphasizing both the potential benefits and limitations of social network capital in addressing persistent poverty.

Here, we construct an undirected social network consisting of *N* = 1225 agents, such that each agent possesses some initial amount of wealth drawn from a normal distribution centred at *μ* = 10 with standard deviation *σ* = 1. An edge is added between two agents with respective wealth levels *w*_*i*_ and *w*_*j*_ with probability

1 $${p}_{ij}=\frac{1}{1+{[{b}^{-1}d({w}_{i},{w}_{j})]}^{\alpha }},$$

where *d*(*w*_*i*_, *w*_*j*_) = ∣*w*_*i*_ − *w*_*j*_∣, *b* is the characteristic distance at which *p*_*ij*_ = 0.5, and *α* is the homophily parameter (a higher value means that attachment is more likely between agents with similar wealth levels) (Talaga and Nowak, 2020). In practice we set the characteristic distance, based on preliminary results, to the average pairwise (wealth) distance between all agents divided by 15.

**Fig. 1:**
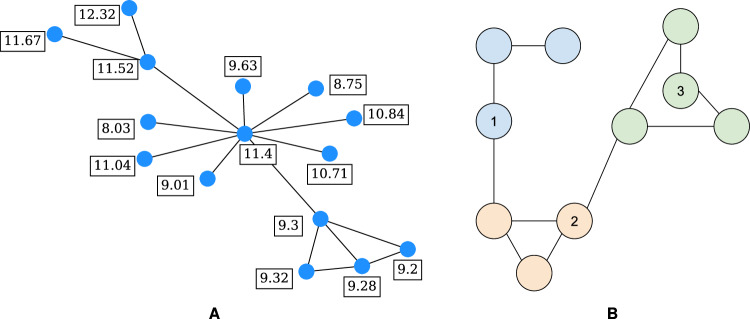
Creation of social network and community. **A** SDA graph with homophily parameter *α* = 8 for 15 agents with wealth values drawn from ${\mathcal{N}}(\mu =10,\sigma =1)$. **B** Toy example of community detection via label propagation. Colors correspond to the result of applying the label propagation algorithm. However, as described in this section, we consider an agent to be a member of its own community as well as a member of any community that agents that it is adjacent to are part of. Hence, node 1 belongs to the blue and orange communities, node 2 belongs to the orange and green communities, and node 3 belongs only to the green community.

Due to the probabilistic nature of edge creation, this algorithm may yield multiple disconnected components. If so, we ensure connectedness as follows: (1) identify the largest connected component and (2) for every other connected component, pick a random node and insert an edge between this node and whichever node inside the largest connected component is closest in wealth. Figure 1a provides an example of a small SDA graph including agent wealth levels to highlight the way in which edges are formed based on the property of homophily. The reader may refer to Fig. 3 of Appendix C for some key distributions (e.g., number of communities, community sizes) for the SDA graphs that were generated with *N* = 1225 agents.

### Agent community

The formation of communities based on homophily is one of the most important characteristics of social networks. In our model, a community is defined as a subset of nodes in a graph such that “connections between those nodes are denser than connections with the rest of the network”, and community structure significantly impacts the types of interactions and dynamics that can occur in a population (Radicchi et al., 2004). Concretely, agents within the same community typically interact and share information more often than with agents in different communities, which has important consequences for how a system evolves over time. Extracting a network’s community structure is therefore an important problem, which we tackle by using a semi-synchronous label propagation algorithm proposed by Cordasco and Gargano (Cordasco and Gargano, 2011). This approach was chosen as it relies on how information diffuses throughout a network to identify communities, is efficient, and shown to always converge to a stable labelling (Cordasco and Gargano, 2011).

Running the algorithm after graph construction yields a set *C* of communities, where each element is a set of agents and such that one agent cannot be in more than one community. However, we expand the notion of community membership as follows: an agent is a member of its own community as well as any community that agents it is adjacent to are part of. Figure 1b displays a stylised example of this procedure. Node 1 belongs to the blue community and the orange community since one of its neighbours is orange. Similarly, node 2 belongs to the orange and green communities. Node 3, however, only belongs to the green community.

### Agent and community resource dynamics

We allow agents of the same community to undertake joint ventures by investing in risky projects, thereby forming self-financing groups, similarly to the work by Martines et al (Gonzales Martinez et al., 2021). Each community is assigned a risky project with two possible outcomes and probabilities summing to one, and an agent can only invest in projects assigned to communities that it is a member of. This assumption reflects real-world constraints often faced in developing economies, where investment opportunities may be localized due to: limited information flows, the importance of trust within known social circles, and the geographical specificity of ventures (e.g., community-based agricultural projects) (Dupont and Roy, 2023; Fafchamps, 2003; Uphoff et al., 1998). While a simplification of globalized interconnected settings, it allows us to focus on the impact of local network structures and community-level dynamics on investment opportunities and resultant economic outcomes.

**Fig. 2:**
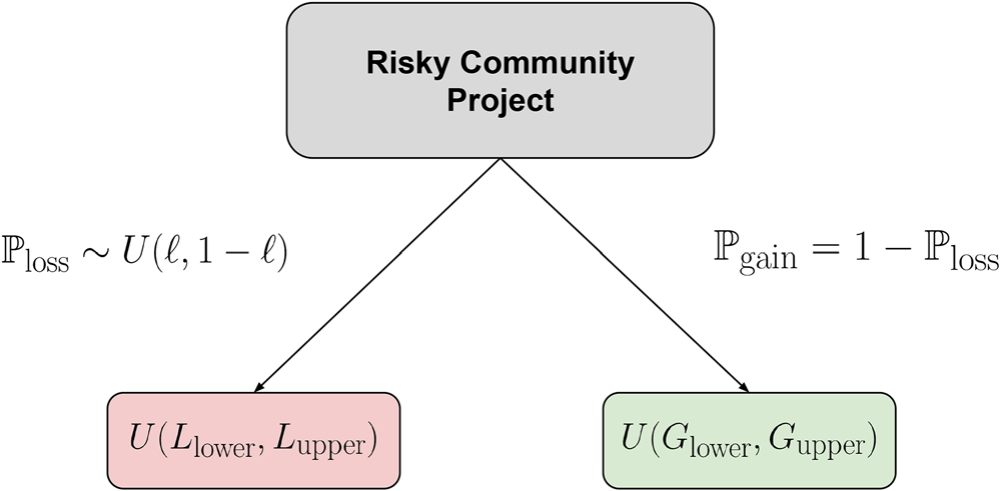
Diagram of a risky community project. ${{\mathbb{P}}}_{{\rm{loss}}}$ is independently drawn once for each project, as are the random loss and gain returns in the red and green boxes, respectively.

A risky project is generated as follows. First, we randomly draw the probability that the project yields a loss from the uniform distribution *U*(*ℓ*, 1 − *ℓ*) with parameter *ℓ* ∈ [0.30, 0.45]. Let us call this probability ${{\mathbb{P}}}_{{\rm{loss}}}$. The probability that the project yields a gain is therefore ${{\mathbb{P}}}_{{\rm{gain}}}=1-{{\mathbb{P}}}_{{\rm{loss}}}$. Finally, we must determine the actual values of the loss and gain. The loss is drawn once per project from uniform distribution *U*(*L*_lower_, *L*_upper_) and the gain from uniform distribution *U*(*G*_lower_, *G*_upper_), with the following relations: *L*_lower_ < *L*_upper_ < 1 < *G*_lower_ < *G*_upper_. Figure 2 illustrates a risky project, including branch probabilities and outcomes.

In addition to community projects, each agent has the option to invest in a safe asset with a guaranteed gain of *G*_safe_ > 1. However, we still wish for projects to be attractive alternatives to the safe asset, so care is taken in choosing parameter values such that the expected return of each project is at least *G*_safe_. Lastly, a project is allowed to take place if and only if the total pooled investment towards that project is at least *θ* ∈ (0, 1) multiplied by the total initial wealth of agents able to contribute towards the project. If this requirement is not met, all invested funds are lost. Otherwise, the project returns the gain with probability ${{\mathbb{P}}}_{{\rm{gain}}}$ and the loss with probability ${{\mathbb{P}}}_{{\rm{loss}}}$.

### Portfolio optimisation

Agents must decide how they wish to apportion their capital between the safe asset and whatever risky projects are available to them (De Bruijn and Antonides, 2022; Haushofer and Fehr, 2014). We let the portfolio of agent *i* be denoted ***P***_*i*_, which is a vector of weights summing to one. Note that ***P***_*i*_ is at least two-dimensional since every agent can invest in the safe asset and is a member of at least one community with a corresponding project.

In order to select a portfolio, agents must perform portfolio optimisation. This problem is approached using Cumulative Prospect Theory (CPT), which was developed in order to better explain how humans make decisions, particularly under uncertainty. The following theoretical overview of CPT closely parallels that of (Luxenberg et al., 2024)

**Fig. 3:**
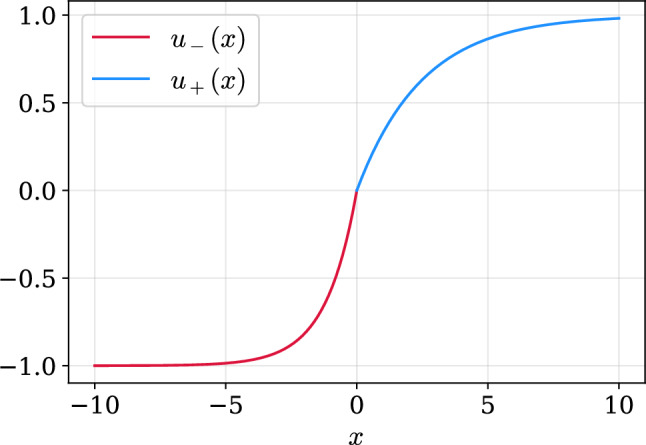
Prospect theory utility function. Parameters: *γ*_−_ = 0.85 (risk attitude for losses) and *γ*_+_ = 0.4 (risk attitude for gains).

Agents are assumed to be risk-averse for gains. However, they are also risk-seeking for losses. For a given return *x*, the prospect theory utility function is given by

2 $${u}^{{\rm{prosp}}}(x)=\left\{\begin{array}{ll}{u}_{+}(x)\quad &x\ge 0\\ {u}_{-}(x)\quad &\,\text{otherwise}\,\end{array}\right.,$$

where ${u}_{+}(x)=1-{e}^{(-x{\gamma }_{+})}$ and ${u}_{-}(x)={e}^{(x{\gamma }_{-})}-1$. Here, *γ*_+_ and *γ*_−_ are parameters shaping the utility function for gains and losses, respectively, reflecting different risk attitudes. There is the additional requirement that *γ*_−_ > *γ*_+_ > 0, which translates to loss aversion. Figure 3 provides a visual representation of Equation (2). The steeper exponential curve for losses (in red) can be interpreted as how a small decrease in wealth would decrease the utility more than an equal increase in wealth would increase utility. In other words, losses are felt more heavily than gains.

Next, we define some empirical distribution of returns for *n* assets. We denote these returns *r*_1_, *r*_2_, …, *r*_*N*_ where ${r}_{j}\in {{\mathbb{R}}}^{n}$. That is, each *r*_*j*_ is a vector of *n* realized asset returns. Now consider some portfolio $p\in {{\mathbb{R}}}^{n}$, such that all weights sum to one. The resulting portfolio returns would be ${r}_{1}^{\top }p,{r}_{2}^{\top }p,\ldots ,{r}_{N}^{\top }p$ where ${r}_{j}^{\top }p\in {\mathbb{R}}$.

Now let *N*^−^ denote the number of negative portfolio returns, and let *N*^+^ denote the number of non-negative portfolio returns, with *N* = *N*^+^ + *N*^−^. After sorting all of the portfolio returns, we can write the following relations:

$${p}^{\top }{\rho }_{1}\le \ldots \le {p}^{\top }{\rho }_{{N}^{-}} < 0\le {p}^{\top }{\rho }_{{N}^{-}+1}\le \ldots \le {p}^{\top }{\rho }_{N},$$

where *ρ*_*i*_ denotes the *i*th portfolio return after sorting. In other words, *p*^⊤^*ρ*_1_ is the biggest loss, and *p*^⊤^*ρ*_*N*_ the biggest gain. Consider also the following probability weighing functions, where *δ*_+_ and *δ*_−_ are parameters between 0 and 1 that capture how agents subjectively distort objective probabilities:

$$\begin{array}{rcl}{w}_{+}(p)&=&\frac{{p}^{{\delta }_{+}}}{{({p}^{{\delta }_{+}}+{(1-p)}^{{\delta }_{+}})}^{1/{\delta }_{+}}}\\ {w}_{-}(p)&=&\frac{{p}^{{\delta }_{-}}}{{({p}^{{\delta }_{-}}+{(1-p)}^{{\delta }_{-}})}^{1/{\delta }_{-}}}\end{array}$$

Using these, we define the following positive and negative decision weights:

$$\begin{array}{rcl}{\pi }_{+,j}^{{\prime} }&=&\left\{\begin{array}{ll}{w}_{+}\left(\frac{{N}^{+}-j+1}{N}\right)-{w}_{+}\left(\frac{{N}^{+}-j}{N}\right)\quad &j=1,\ldots ,{N}^{+}-1\\ {w}_{+}\left(\frac{1}{N}\right)\quad &j={N}^{+},\end{array}\right.,\\ {\pi }_{-,j}^{{\prime} }&=&\left\{\begin{array}{ll}{w}_{-}\left(\frac{{N}^{-}-j+1}{N}\right)-{w}_{-}\left(\frac{{N}^{-}-j}{N}\right)\quad &j=1,\ldots ,{N}^{-}-1\\ {w}_{-}\left(\frac{1}{N}\right)\quad &j={N}^{-}\end{array}\right..\end{array}$$

We wish for ${\pi }_{+}^{{\prime} }$ and ${\pi }_{-}^{{\prime} }$ to be monotonically increasing, and thereby assign greater weight to extreme outcomes. Although this is the case for most parameter choices, some can lead to the violation of this desirable property. We can ensure that ${\pi }_{+}^{{\prime} }$ and ${\pi }_{-}^{{\prime} }$ are non-decreasing by substituting ${\pi }_{+,j}^{{\prime} }$ with $\min (\underset{+}{\overset{{\prime} }{\pi }})$ for all $j < \,\text{argmin}\,({\pi }_{+}^{{\prime} })$, and substituting ${\pi }_{-,j}^{{\prime} }$ with $\min (\underset{-}{\overset{{\prime} }{\pi }})$ for all $j < \,\text{argmin}\,({\pi }_{-}^{{\prime} })$.

In order for ${\pi }_{+}^{{\prime} }$ and ${\pi }_{-}^{{\prime} }$ to each have length *N* instead of *N*^+^ and *N*^−^ respectively, we zero-pad both sequences: ${\pi }_{+}=({{\boldsymbol{0}}}^{{N}_{-}},{\pi }_{+}^{{\prime} })$ and ${\pi }_{-}=({{\boldsymbol{0}}}^{{N}_{+}},{\pi }_{-}^{{\prime} })$, where ***0***^*k*^ denotes a zero-vector of dimension *k*.

Let us also define the following two functions operating element-wise on some vector *x*:

$$\begin{array}{rcl}{\phi }_{+}(x)&=&\max (0,x)\\ {\phi }_{-}(x)&=&-\min (0,x)\end{array}$$

Using these, we can finally write the CPT utility function as follows:

3 $${U}^{{\rm{cpt}}}(p)={f}_{{\pi }_{+}}({\phi }_{+}({u}_{+}({\boldsymbol{R}}p)))-{f}_{{\pi }_{-}}({\phi }_{-}({u}_{-}({\boldsymbol{R}}p))),$$

where ***R*** is the matrix of empirical asset returns and *f*_*π*_ is the weighted-ordered-sum function, defined as

$${f}_{\pi }(x)=\mathop{\sum }\limits_{i=1}^{N}{\pi }_{i}{x}_{(i)},$$

where *x*_(*i*)_ denotes the *i*th smallest element of vector *x* and *π* is assumed to be a monotonically increasing probability vector, which we have ensured is the case.

The CPT optimisation problem to be solved by agents is thus to find a portfolio *P* maximising the CPT utility:

4 $$\mathop{\max }\limits_{P}\,\,{U}^{{\rm{cpt}}}(P),$$

which is non-convex as shown by (Luxenberg et al., 2024). Further, Luxenberg et al. (2024) show that CPT utility can be decomposed as a difference of two functions. The first term is a convex function with concave arguments, and the second term is a convex function with convex arguments. This structure allows us to derive maximum CPT utility (Luxenberg et al., 2024).

In practice, we assign each agent its own CPT utility function. The key CPT parameters for each agent are: *γ*_+_ (risk attitude for gains), *γ*_−_ (risk attitude for losses), *δ*_+_ (probability weighting for gains), and *δ*_−_ (probability weighting for losses). These parameters are drawn from uniform distributions: ${\gamma }_{+} \sim U({\gamma }_{+}^{{\rm{lower}}},{\gamma }_{+}^{{\rm{upper}}})$, ${\gamma }_{-} \sim U({\gamma }_{-}^{{\rm{lower}}},{\gamma }_{-}^{{\rm{upper}}})$, ${\delta }_{+} \sim U({\delta }_{+}^{{\rm{lower}}},{\delta }_{+}^{{\rm{upper}}})$, and ${\delta }_{-} \sim U({\delta }_{-}^{{\rm{lower}}},{\delta }_{-}^{{\rm{upper}}})$.

### Portfolio update and attention mechanism

An agent does not keep the same portfolio throughout the simulation. Agents learn and adjust their strategies over time based on observed payoffs or shocks, leading to dynamic equilibria that reflect real-world fluctuations. Initially, we generate *M* random returns for each risky project as well as the safe asset (though these are constant), and each agent *i* bases its initial portfolio ***P***_*i*,initial_ on this data. However, as projects yield returns at each time step, agents can use this information to update their portfolio choices. For example, an agent may decide to reduce the amount invested towards a project that keeps failing and instead redistribute these funds towards other, more promising projects or even the safe asset.

This is done using an attention mechanism as follows. Each agent *i* is initialised with an independent attention parameter *a*_*i*_ randomly drawn from the uniform distribution *U*(0, 1). Then, anytime an agent updates its portfolio throughout the simulation, it computes the following weighted sum:

5 $${{\boldsymbol{P}}}_{i}=(1-{a}_{i})\,{{\boldsymbol{P}}}_{i,{\rm{initial}}}+{a}_{i}\,{{\boldsymbol{P}}}_{i,{\rm{observations}}},$$

where ***P***_*i*,observations_ is the optimal portfolio of agent *i* based on observed project returns since the start of the simulation. Hence, the higher an agent’s attention, the more importance it will place on the project returns it observes during the simulation when choosing a portfolio. Portfolio update times are Poisson-distributed with parameter *λ*, and each agent has independently sampled update times. Furthermore, we only allow for updates to be carried out after an initial warm-up period of 5 model steps.

### Consumption

At each time step *t*, each agent *i* must consume a portion *c*_*i*,*t*_ of its current wealth *w*_*i*,*t*_. The amount consumed is determined by the saving propensity parameter *β* ∈ (0, 1), and is calculated as *c*_*i*,*t*_ = 1 − *β* ⋅ *w*_*i*,*t*_. Once capital has been consumed, the remainder is invested according to the agent’s current portfolio, and the agent’s wealth at the next time step is the return on investments (both safe and risky).

### Summary of model parameters

Table 1 summarises key model parameters. Parameters that we wish to vary for the sensitivity analysis have their values listed in square brackets, denoting uniform intervals. Other parameters have fixed values for all simulations.

**Table 1:** Summary of agent-based model parameters.

Parameter	Description	Value(s)
*N*	Number of agents	1225
*K*	Number of model steps	100
*λ*	Poisson parameter for generating update times	10
*μ*	Average of initial wealth distribution	10
*σ*	Standard deviation of initial wealth distribution	1
*M*	Initial number of project returns	2000
*L*_lower_	Lower uniform bound for project loss	0.90
*L*_upper_	Upper uniform bound for project loss	0.95
*G*_lower_	Lower uniform bound for project gain	1.60
*G*_upper_	Upper uniform bound for project gain	[1.70, 8.00]
*G*_safe_	Safe asset gain	1.10
*ℓ*	Parameter for generating project loss probabilities	[0.30, 0.45]
*θ*	Minimum required project investment	[0.05, 0.95]
*β*	Saving propensity	[0.70, 0.80]
*α*	Homophily parameter	[2.00, 12.00]
*b*	Characteristic distance	See “Agent social network”
${\gamma }_{+}^{{\rm{lower}}}$	Lower uniform bound for *γ*_+_ (risk attitude for gains)	5
${\gamma }_{+}^{{\rm{upper}}}$	Upper uniform bound for *γ*_+_ (risk attitude for gains)	30
${\gamma }_{-}^{{\rm{lower}}}$	Lower uniform bound for *γ*_−_ (risk attitude for losses)	31
${\gamma }_{-}^{{\rm{upper}}}$	Upper uniform bound for *γ*_−_ (risk attitude for losses)	70
${\delta }_{+}^{{\rm{lower}}}$	Lower uniform bound for *δ*_+_ (weighting parameter for gains)	0.50
${\delta }_{+}^{{\rm{upper}}}$	Upper uniform bound for *δ*_+_ (weighting parameter for gains)	0.70
${\delta }_{-}^{{\rm{lower}}}$	Lower uniform bound for *δ*_−_ (weighting parameter for losses)	0.71
${\delta }_{-}^{{\rm{upper}}}$	Upper uniform bound for *δ*_−_ (weighting parameter for losses)	0.90

Algorithm 1 summarises the key steps of the agent-based model. All parameters are fixed except five, which are listed at the top of the algorithm as input parameter set. After initializing global attributes such as the network, communities, projects, and random initial returns, we initialize individual attributes for *N* = 1225 agents. Then, we perform *K* = 100 model steps during each of which agents consume some capital, invest the rest according to their portfolio, and may also update their portfolio. Before going to the next step, project returns are divided amongst investors to compute each agent’s wealth for the next time step. At the end of the simulation, we have an *N* × *K* matrix of agent wealth trajectories and knowledge of agent attributes, project returns, etc. *K* = 100 model steps were found to be sufficient during preliminary experiments for aggregate statistics to stabilize and reach a steady state.

#### Algorithm 1

**Agent-based model for multi-level poverty trap formation** 

### Experiments

Saltelli sampling is used to generate different combinations of parameters (Saltelli, 2002). We choose to have 1024 unique values per parameter, resulting in a total of 7168 parameter combinations. Furthermore, twenty repetitions with different random seeds are carried out for each parameter combination to account for stochastic effects. This approach avoids dynamically altering parameters during simulation runs, instead focusing on pre-generated sample sets for systematic analysis. It is particularly valuable for identifying key drivers of variability in models with high-dimensional parameter spaces.

In addition to studying the resulting wealth trajectories of individuals and communities, we also examine whether a one-time capital injection can help the poorest agents escape poverty—mobility in this context can be understood to mean the capacity of individuals to escape poverty when receiving assets. In order to do this, we randomly select one parameter combination from the All Poor regime and one from the Some Rich regime. Then, for each one, we let our model run for 100 time steps, at which point we give 10 units of capital to the 100 poorest agents. Subsequently, we allow the model to run for an additional 100 steps and examine whether the targeted agents have managed to avoid falling into poverty. 20 repetitions are performed to account for stochastic effects. Lastly, we study the model’s sensitivity to parameters using a global sensitivity analysis method (Bazyleva et al., 2023, 2024) to compute diffusion coordinates and sensitivity indices, shown in Figs. 1 and 2 of Appendix B, respectively. The appendix also contains additional details regarding the method used.

## Results

### Transition from extreme poverty to extreme inequality

We identify three distinct regimes of model behaviour, representing characteristic steady state outcomes, based on the wealth trajectories of agents across different parameter combinations and repetitions. In the first regime, which we label All Poor, every agent in the population has a lower wealth by the end of the simulation compared to their starting wealth. This regime is characterised by extreme poverty, and agents are unable to avoid or mitigate financial losses. In the second regime, the Some Rich regime, one or more agents have a final wealth greater than their initial wealth. Most of the time, this wealth accumulation is experienced by only a small minority, thereby giving rise to significant inequality and wealth disparity. Finally, in the third regime, All Rich, every agent has a final wealth greater than their initial wealth. We observe the following proportions of simulations that land in each regime: 12.24% (All Poor), 87.7% (Some Rich), and 0.06% (All Rich). It is worth noting that the All Poor and Some Rich regimes correspond very closely with single and double equilibrium poverty traps, as studied in depth by (Barrett and Carter, 2013) Although formal fitting to specific parametric wealth distribution models was not carried out in this study, we wished to validate the steady-state wealth distributions generated by our model, particularly for the more realistic Some Rich regime. Therefore, we computed the normalized Complementary Cumulative Distribution Function (CCDF) for our steady-state wealth distributions. As illustrated in Fig. 8 (Appendix H), a sample of these simulated CCDFs was compared against an empirical CCDF derived from the UK wealth distribution, with wealth values normalized for appropriate comparison. The figure demonstrates a good alignment between our simulated wealth distributions and the empirical data, suggesting that our model is able to capture key characteristics of real-world wealth inequality, especially in its general shape and tail behaviour.

**Fig. 4:**
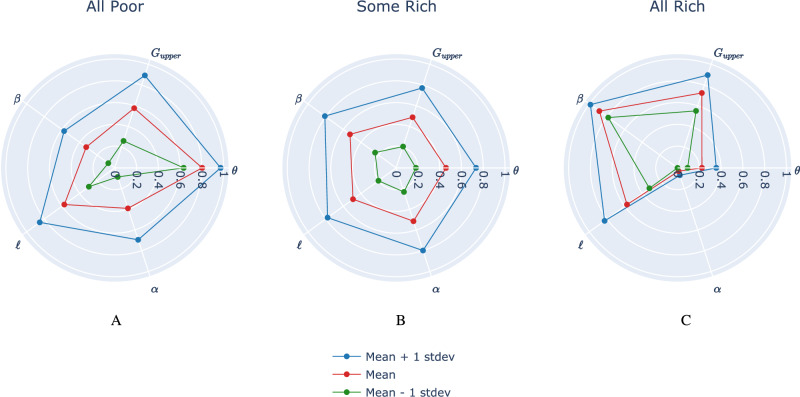
Average Scaled Parameter Profiles Across Wealth Regimes. Average parameter values (in red) resulting in each of the three distinct regimes: All Poor, Some Rich, All Rich. Key parameters displayed are: *θ* (minimum project investment threshold), *G*_upper_ (upper bound for project gain magnitude), *β* (saving propensity, where a lower value means more consumption), *ℓ* (probability of project loss), and *α* (homophily in social network formation, where a higher value means more initial wealth-based segregation). Parameter values have been independently scaled between 0 and 1 based on the ranges defined in Table 1. In blue and green are one standard deviation above and below the average parameter values, respectively. **A** Parameter values giving rise to the All Poor regime. High values of homophily (*α*) and project cost (*θ*) are likely to yield this regime. **B** Parameter values giving rise to the Some Rich regime. **C** Parameter values giving rise to the All Rich regime. Homophily and project cost are notably smaller for simulations landing in this regime.

Our findings show that the evolution of poverty and inequality (state space) is driven by various social, economic, and financial structures (represented by the parameter space of the model) and can explain the observed transition from extreme poverty to pervasive inequality. Figure 4 illustrates the typical parameter ranges giving rise to each of the three regimes. The influential parameters were identified using global sensitivity analysis, as described in Appendix B (see Figs. 1 and 2). The scale of each parameter is adjusted using min-max normalization such that 0 corresponds to the minimum possible value of that parameter, and 1 corresponds to its maximum value (see Table 1 for a summary of model parameters). For each regime, the vertices along the red polygon correspond to average parameter values giving rise to that regime. The analysis of the parameter space reveals three key insights for reducing poverty and inequality.

First, parameter *θ*, which modulates the minimum project cost required of each community in order for a project to be carried out, plays a significant role in shaping model behaviour. On average, *θ* is close to 0.8 of its maximum value in the All Poor regime (implying that project costs are high), whereas it is only 0.45 for the Some Rich regime, and 0.2 for the All Rich regime. Clearly, reducing the cost of undertaking community projects is helpful in lowering the barrier to financial well-being and tends to increase the likelihood of agents avoiding poverty. This indicates that reducing frictions related to financial inclusion can increase the level of entrepreneurship, allowing financially constrained individuals to become entrepreneurs and move out of poverty. At the aggregate level, these effects could ultimately boost economic activity, reduce poverty, and potentially increase income equality—as observed in previous empirical studies (Omar and Inaba, 2020; Park and Mercado, 2015).

The parameter *ℓ*—which determines the probability of projects yielding a loss rather than a gain—does not appear to be different across regimes, while the upper uniform bound for the magnitude of project gains, *G*_upper_, is on average significantly higher for the All Rich regime. This implies that increasing project returns is more favourable to agents than increasing the frequency of project success. Second, the saving propensity parameter *β* also plays an important role in giving rise to the different regimes. In the All Poor regime, *β* typically takes lower values, indicating that agents consume more and invest less towards risky projects. As pointed out by Banerjee and Duflo, a “main challenge for the poor who try to save is to find safety and a reasonable return” (Banerjee and Duflo, 2007). Indeed, in the All Poor regime, projects tend to fail more often than not, making it difficult for agents to invest their savings. However, agents in the All Rich regime tend to save more of their wealth in order to invest it towards high-return projects, which tend to be successful. This is in line with traditional perspective on micro and macroeconomic growth, which posits that increased savings, when channelled into investment, would fuel economic growth in households (Steinert et al., 2018). Several empirical studies show that higher savings rates not only directly boost investment but also indirectly increase steady-state output, as rising savings often correlate with rising income, thus stimulating further investment (Do, 2023; Karlan et al., 2014). Lastly, we find that the homophily parameter *α* must take on particularly low values in order for the All Rich regime to emerge. This translates to lower homophily, implying that there is less initial economic segregation based on wealth, thereby improving the chances of poorer agents to be included in community projects with higher investments and chance of success. By contrast, in the Some Rich regime, homophily tends to be higher, which implies more segregation and leads to greater eventual inequality. The share of high-socioeconomic status (SES) friends among individuals with low SES (low homophily)—known as economic connectedness—is among the strongest predictors of upward income mobility (Chantarat and Barrett, 2012; Chetty et al., 2022; Pena-López et al., 2021; Tóth et al., 2021). A recent study identified that people endowed with high levels of economic and human capital enjoy improved accessibility and networks with a high prevalence of instrumental relations (Pena-López et al., 2021). There is essential inequality in the endowment of social capital, which augments economic inequality. Further, when inequality is socially embedded, traditional re-distributive policies demonstrate limited effectiveness (Pena-López et al., 2021).

Beyond individual and distributional outcomes, aggregate wealth dynamics differ across the three regimes. The All Poor regime is characterised by a net destruction of aggregate wealth over time since projects consistently fail or yield low returns, and consumption erodes capital. Conversely, the Some Rich regime demonstrates the model’s capacity for aggregate wealth accumulation, even if wealth becomes highly concentrated among a minority of agents. The rare All Rich regime shows robust growth in both individual and aggregate wealth. This capacity for aggregate wealth generation in the Some Rich and All Rich regimes is crucial, as it theoretically provides the resources that could be targeted by redistributive interventions, although the effectiveness of such interventions depends on the underlying systemic factors, as explored in “Asset-based interventions are context dependent”. Nonetheless, Fig. 4 indicates that growth, in terms of total wealth, is most likely when project cost (*θ*) and homophily (*α*) are low, and saving propensity (*β*) is high.

**Fig. 5:**
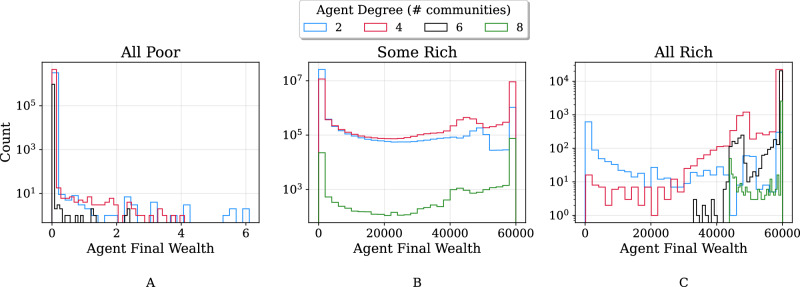
Effect of access to communities on economic prosperity. **A** Distribution of final agent wealth for All Poor regime simulations. Colours correspond to the number of communities that an agent is part of. In this case, a lower degree seems to be indicative of higher final wealth. **B** Same as sub-figure A but for the Some Rich regime. The dependence of final wealth on agent degree is less evident. **C** Same as sub-figures A and B, but for the All Rich regime. In this case, a higher degree appears to be associated with higher final wealth.

Figure 5 illustrates the relationship between the final wealth of agents in each regime and the number of projects available to the agents (the community degree). Interestingly, although all agents tend towards poverty, it seems favourable in the All Poor regime to be part of fewer communities (and thus investment projects). This can be seen from the tails of the black, red, and blue distributions extending further to the right as community degree decreases, respectively. This story is completely flipped in the All Rich regime, where a higher community degree appears to lead to a higher final wealth. Given that projects, on average, are successful more often in the “All Rich” regime, it indeed makes sense for an agent to diversify their investments. By contrast, because projects fail often in the All Poor regime (for instance, due to higher *θ* values), agents tend to invest in fewer projects, thereby taking fewer risks and maintaining a larger investment towards the safe asset. This pattern of behaviour is in fact supported by a growing body of literature. For instance, experimental work by Yesuf and Bluffstone with poor households in Ethiopia reveals a high degree of risk aversion and risk-averting behaviour with “perhaps significant implications for long-term poverty” (Yesuf and Bluffstone, 2009). Indeed, farmers are often prone to sub-optimal decision making due to being less willing to undertake projects with high expected returns, which results from near constant exposure to risk factors in their daily work (Rosenzweig and Binswanger, 1993).

**Fig. 6:**
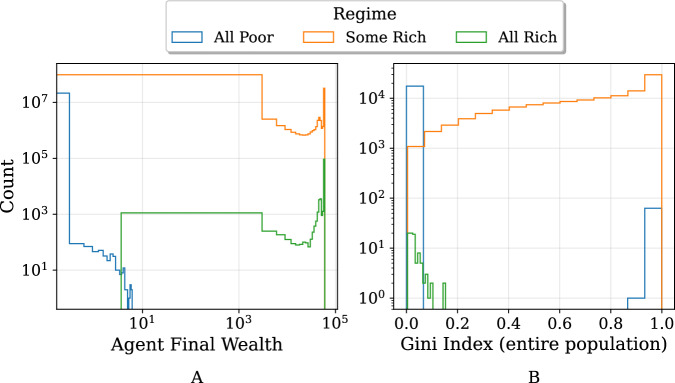
Comparison of final agent wealth distributions and Gini indices. Data have been aggregated from all simulation runs (7168 parameter combinations × 20 repetitions each), with each run categorized into the All Poor, Some Rich, or All Rich regime based on final agent wealths. **A** Final wealth of agents in the All Poor regime never surpass small values, whereas agents in the Some Rich regime can attain high levels of wealth. Meanwhile, in the All Rich regime, final wealth levels close to zero are, by definition, impossible. **B** Comparison of Gini indices (at final time step) for populations in different regimes. The All Poor and All Rich regimes tend to exhibit low Gini indices (signifying low inequality), whereas the Some Rich regime exhibits mostly high Gini indices and therefore inequality.

Figure 6 displays the aggregated final agent wealth distributions for each regime, as well as corresponding Gini indices (calculated at the end of each simulation at the population level). While agent wealth levels quickly taper off in the All Poor distribution, both of the Some Rich and All Rich distributions have a tail of very rich agents at the far right. Moreover, the All Rich distribution does not have any data points at or near zero since agents have all attained a higher wealth level than their starting point.

In the All Poor and All Rich regimes, Gini coefficients are understandably very low (with a few exceptions in the All Poor regime), since there is little inequality due to all agents being either poor or rich as regime names indicate. However, the intermediate Some Rich regime is characterised by high levels of inequality, with Gini coefficients generally taking on values greater than 0.8. Further details on the relationship between total population wealth and the Gini index for the Some Rich regime may be found in Fig. 5 of Appendix E. The reader may also refer to Fig. 4 of Appendix D to examine the distributions of project returns by regime. While the Gini coefficient provides a useful summary measure of inequality, analysing the full wealth distribution offers further insights. For instance, in representative Some Rich simulations, the top 10% of agents frequently hold well over 80–90% of the total wealth, underscoring the high concentration of wealth suggested by the Gini coefficient. Future work could further explore alternative metrics in order to better capture such nuances.

Overall, the transition from extreme poverty to extreme inequality found here is reminiscent of the global historical trend presented in the Introduction, wherein we argue that many nations have succeeded in reducing poverty over the last decades at the cost of increased inequality.

### Emergence of multi-level poverty traps

Similar to our definition of regimes for individual wealth trajectories, we may also define regimes at the community level as follows. In the All Poor regime, each community’s final total wealth is less than its total initial wealth. Meanwhile, in the Some Rich regime, some communities have final total wealth greater than total initial wealth, and all communities have higher final total wealth in the All Rich regime.

**Fig. 7:**
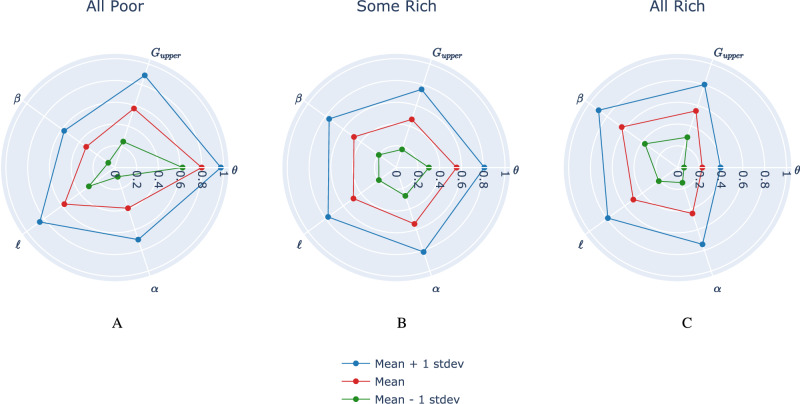
Average parameter values giving rise to different regimes at the community level. Key parameters displayed are: *θ* (minimum project investment threshold), *G*_upper_ (upper bound for project gain magnitude), *β* (saving propensity, where a lower value means more consumption), *ℓ* (probability of project loss), and *α* (homophily in social network formation). Parameter values have been independently scaled between 0 and 1 based on the ranges defined in Table 1. In blue and green are one standard deviation above and below the average parameter values, respectively. **A** High values of project cost (*θ*) are likely to yield the All Poor regime. **B** Parameter values for the Some Rich regime. **C** Parameter values for the All Rich regime at the community level. The effect of homophily is not as pronounced as it was for the individual-level regimes (see Fig. 4). Higher values of consumption (*β*) appear more likely for the All Rich regime compared to the Some Rich and All Poor community-level regimes.

After categorizing all model runs into these community-level regimes, we construct another radar plot similar to Fig. 4 in order to examine the parameter ranges giving rise to each regime. Figure 7 displays these results. The proportions of runs for each regime are as follows: 12% (All Poor), 62% (Some Rich), and 26% (All Rich). Interestingly, we observe a much higher proportion of runs landing in the All Rich regime at the community level compared to the individual level regime definition. The All Poor and Some Rich radar plots look quite similar to those of Fig. 4, but the All Rich has striking differences. Although project costs (*θ*) still need to take on small values in order for all communities to increase their total wealth over the course of the simulation, we notice that lower homophily (small *α*) is not as strong of a precondition. Furthermore, simulations giving rise to the All Rich community regime tend to have lower values of both *β* (saving propensity) and *G*_upper_ (upper bound for project returns) compared to the All Rich radar plot of Fig. 4.

Interestingly, we identify 229 simulations landing both in the All Poor *community* regime and the Some Rich *individual* regime. This indicates that it is possible for some agents to acquire significant wealth, despite all communities tending towards poverty. Conversely, we also find that as many as 26% of our simulations correspond both to the All Rich community regime and the Some Rich individual regime. This implies that we frequently find societies where all communities have a high total final wealth despite only some agents accumulating wealth. In other words, while the total wealth of communities can significantly increase over the course of a simulation, it can also be the case that many agents remain significantly impoverished. Table 2 summarises the (joint) number of simulations landing in individual and community-defined regimes.

**Table 2:** Joint number of simulations landing in individual and community-defined regimes.

Community regime	Individual regime	Count
All poor	All poor	17,537
All poor	Some rich	229
All poor	All rich	0
Some rich	All poor	0
Some rich	Some rich	88,111
Some rich	All rich	0
All rich	All poor	0
All rich	Some rich	37,396
All rich	All rich	87

Overall, we conclude that our model gives rise to poverty traps at three distinct levels. One such trap is clearly at the *macroscopic* scale, where the population as a whole tends towards poverty. Interestingly, we also observe a high number of simulations where only some agents become rich and others do not, hinting at high levels of inequality on a global scale. Another set of traps is at the *meso* scale (community). We find simulations where communities tend towards poverty, but have a few rich community members able to avoid poverty. We also have communities tending towards poverty, with all community members also being poor. Finally, there are mechanisms of traps at the *micro* (individual) scale—we find simulations where communities are overall rich, but some community members are stuck in poverty. In fact, as mentioned earlier, as many as 62% of simulations give rise to societies where some communities thrive and others do not. We thus find that inequality and poverty traps can arise at the population level, but also within and between communities. This result is consistent with many empirical findings, in particular work by Barrett et al., where the authors argue that poverty traps can appear not only at the individual or household level, but also at community, regional, and even national scales (Barrett and Carter, 2013). This multilevel poverty trap approach offers a distinct advantage by enabling an effective allocation of resources for interventions at the most appropriate levels. By understanding how interventions at different levels can have cascading effects, we can ensure targeted and coordinated efforts. Unlike relying on ad hoc decisions or anecdotal experiences, stylized modelling facilitates reasoning through various poverty alleviation strategies and outcomes, aiding practitioners in making informed decisions.

### Agent heterogeneity, information poverty, and sub-optimal diversification

In order to isolate and examine the effect of individual differences (attention and CPT utility parameters) between agents, we group agents according to the set of projects that they have access to. For instance, one group of agents could consist of those who have access to community projects 2 and 3, and another group of agents could contain those who have access to community projects 2, 3, and 5. These would be distinct groups, even though all agents in the second group have access to all projects accessible to agents in the first group.

**Fig. 8:**
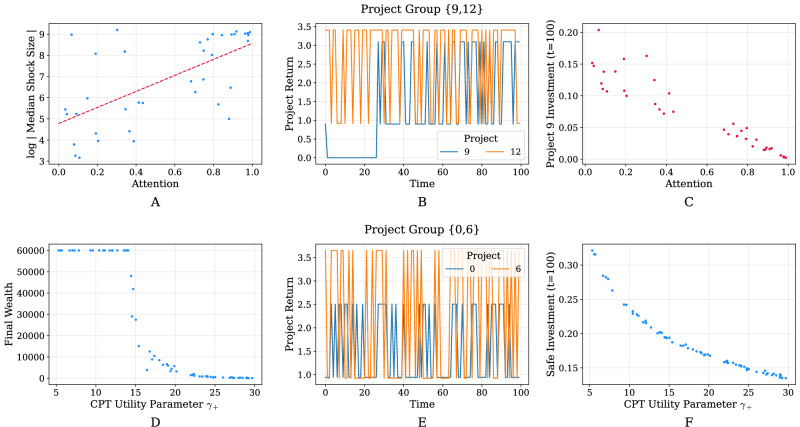
Impact of agent attention and risk attitude towards gains on shocks, final wealth, and investment strategy. Sub-figures A through C show results from a single, hand-picked simulation run that resulted in the Some Rich regime. Sub-figures D through E similarly show results from a different single, hand-picked simulation run resulting in the Some Rich regime. **A** Impact of agent attention on the median shock size experienced for agents with access to the same two community projects (projects 9 and 12). Agents with higher attention experienced economic shocks of greater magnitude. **B** Returns of community projects 9 and 12. **C** Impact of agent attention on the proportion invested in community project 9 by the end of the simulation. **D** Impact of the *γ*_+_ CPT utility parameter (risk attitude for gains, influencing investment choices) on agent final wealth for agents with access to the same two community projects (projects 0 and 6). **E** Returns of community projects 0 and 6. **F** Impact of *γ*_+_ CPT utility parameter (risk attitude for gains) on the proportion invested into the safe asset by the end of the simulation.

Let us first pay attention to the top row of Fig. 8, where we highlight the impact of agent attention on the magnitude of economic shocks (defined as decreases in wealth between subsequent time steps) for agents with access to community projects 9 and 12 in a given simulation. The leftmost figure reveals a positive correlation between attention and the magnitude of economic shocks that agents experience— higher attention translates to experiencing more significant shocks. In order to explain this outcome, we want to better understand how projects 9 and 12 are performing over the course of the simulation. The middle figure reveals that project 9 fails consistently for the first 25 steps or so. Agents with high attention will notice this and invest more capital towards project 12 when updating their portfolio. On the other hand, agents with lower attention will ignore these empirical returns and favor their initial portfolio. The rightmost figure demonstrates this clearly, where we notice that lower attention results in investing a larger proportion of capital towards project 9 by the end of the simulation. This investment, although initially dubious, ends up benefiting agents with low attention since they tend to have more diversified portfolio and are thus able to better absorb economic shocks compared to agents with high attention.

Now let us take a look at the second row of Fig. 8 for an example of how CPT utility parameter *γ*_+_ (which models an agent’s risk attitude towards potential gains) can impact the final wealth of agents with access to projects 0 and 6. The leftmost figure shows a steep transition where agents with *γ*_+_ < 15 are extremely rich by the end of the simulation whereas agents with a value for this parameter greater than 15 converge towards a state of poverty. The central figure shows that both projects 0 and 6 yield nonzero returns throughout the entire simulation. In fact, these empirical returns agree very closely with what we would expect, unlike the previous example, where project 9 failed for numerous subsequent steps. Therefore, in this scenario, attention plays a rather insignificant role when agents update their portfolios. The difference in final wealth is instead attributable to *γ*_+_, and we can observe from the rightmost figure that a lower *γ*_+_ value translates to a higher proportion of wealth being invested in the safe asset. In other words, for this particular group of agents, it is more beneficial to take less risk by investing in the safe asset, presumably due to the risky and volatile nature of the project returns. Although not represented in this rightmost figure, we may even expect that too little risk-taking could end up being harmful (for instance, if the safe investment proportion was as high as >0.70), since agents would not end up benefiting from the high returns of the projects as much.

To conclude this section, we remark that examples such as the ones of Fig. 8 abound throughout the many simulations that were carried out. We have restricted the present analysis in order to showcase the complex and rich behaviour that can be observed using our model as a result of heterogeneity in the agent population. Importantly, whether attention and *γ*_+_ (risk attitude towards gains) are related to economic shocks or final wealth depends on numerous factors, such as which group of agents is considered, but also on which model parameters and regimes are being examined. This outcome highlights the importance of considering traps at multiple levels, since behaviour can vary drastically between different groups or communities of agents even when they are part of the same larger population. Two key insights emerge from our analysis. First, a crucial link may be established between the attention mechanism in our model and the notion of “information poverty” in the literature, which is a situation in which individuals or communities do not have the necessary skills or knowledge to obtain information, as well as to interpret and apply it correctly to a particular problem (Marcella and Chowdhury, 2020). Agents with higher attention pay greater attention to empirical observations, implying that they have better access to information. Similarly, in real-world settings, individuals or communities with greater access to sources of information, such as education or the Internet, are less likely to experience information scarcity, thereby empowering them to make more informed decisions and respond appropriately to different scenarios that may arise. While this analogy is not perfect, it does underscore the importance of what information agents have access to, and crucially, what they decide to do with it: even minor differences in information access and information use can lead to drastically different outcomes for different agents. Second, we show that suboptimal diversification due to information acquisition, particularly in the context of investments or economic activities, can indeed have an impact on poverty. Suboptimal diversification can lead to increased volatility in income (Capuano and Ramsay, 2011; Van Nieuwerburgh and Veldkamp, 2010). Previous research shows that risk-averse individuals tend to focus more on past outcomes (high attention), especially negative ones, when making decisions under uncertainty (Dillenberger and Rozen, 2015; Li et al., 2023). This heightened attention to prior losses or adverse experiences can increase their sensitivity to risk and drive more cautious choices, leading to suboptimal diversification. For example, if a household relies solely on one unstable source of income, such as seasonal agricultural work, it may face periods of scarcity when that income dries (Michler and Josephson, 2017). This can push them into poverty or exacerbate their existing poverty. In times of economic downturns, natural disasters, or other crises, families with sub-optimal diversification may lack resilience to bounce back, potentially slipping into poverty or facing prolonged periods of financial hardship (Antonelli et al., 2022). At the community level, a lack of diversification can lead to widespread unemployment and poverty if, for example, a community heavily relies on a single industry that faces a downturn or technological disruption.

### Asset-based interventions are context dependent

**Fig. 9:**
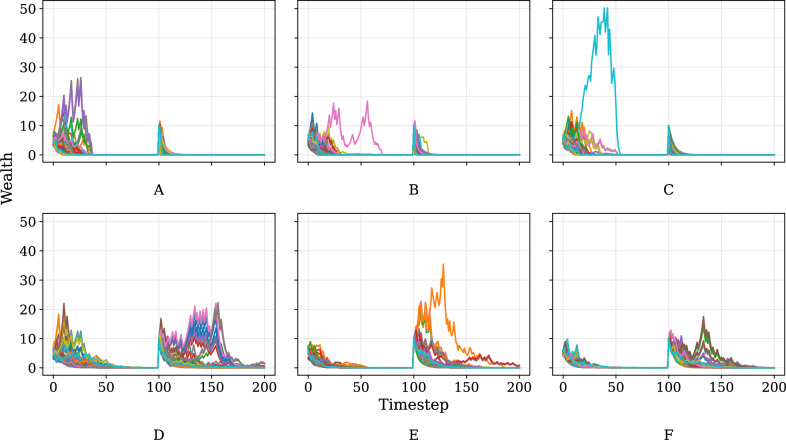
Examples of wealth trajectories for the 100 poorest agents following a one-time capital injection after 100 time steps. **A**–**F** display results from a distinct, single simulation run chosen to illustrate typical outcomes. Three example runs are shown for interventions in the All Poor regime (top row, **A**–**C**) and three for the Some Rich regime (bottom row, **D**–**F**). Interventions were performed for a specific parameter combination representative of each regime, with 20 Monte Carlo repetitions conducted for each; these plots only show illustrative individual repetitions.

Lastly, we turn our attention to the results of intervention experiments, as described in “Experiments”. Figure 9 displays a sample of three out of 20 interventions that were performed for a choice of parameters corresponding to the All Poor (top row) and Some Rich (bottom row) regimes. At *t* = 100, the poorest 100 agents were identified and received a wealth injection of 10 units, after which the model was allowed to run for another 100 time steps. The trajectories displayed below are those corresponding to this subset of agents only.

For the All Poor regime, we find that in all 20 repetitions of our experiments, the targeted agents all fall back into poverty with final wealth approaching zero (<10^−8^) by *t* = 200. This indicates that interventions that do not change the underlying landscape of factors (in this case the parameter space) contributing to poverty will not favour impoverished agents in the long term. By contrast, for the Some Rich regime, approximately 32% of targeted agents manage to escape poverty by *t* = 200, with a standard deviation of 13%. Note that here we define the poverty line as being set by the wealth of the wealthiest agent among the 100 poorest agents at the end of the experiment. This outcome implies that when the underlying parameter space is conducive to upward mobility, a capital injection can prove to be successful at helping agents to escape from poverty.

This contrast in effectiveness between regimes suggests that interventions targeting systemic parameters (like *θ*, the minimum project investment threshold, which represents a barrier to financial inclusion/entrepreneurship) can have more profound and lasting effects than one-time capital injections when the underlying system dynamics are unfavourable. Lowering *θ* fundamentally alters the “rules of the game”, making projects systemically more accessible and viable for all agents within that community over the long term. A cash injection, while potentially enabling an agent to meet the *θ* threshold for one or a few investment cycles, does not change the high cost or low viability of projects if *θ* remains high and other adverse conditions persist. Thus, without systemic changes to opportunity structures, individual boosts may offer only temporary relief in the All Poor regime. It is important to note that our cash transfer intervention was modelled with a fixed magnitude (10 units) and a fixed number of beneficiaries (100 poorest agents); exploring variations in these parameters, such as larger transfers or different targeting strategies, could yield different insights and is a subject for future investigation.

The efficacy of capital injection, or cash transfers, as a means of combating poverty remains a controversial topic even today. A recent article by Miao and Li examining the impact of cash transfers towards rural households in China casts doubt on whether this form of intervention necessarily guarantees positive long-term effects (Miao and Li, 2023). Although the cash transfers enabled poor rural residents to spend more on healthcare, they also led to a decrease in labour supply and no change in education spending, despite education being a significant factor in long-term poverty mitigation. Our results offer a similar point of view, especially for the All Poor regime, in the sense that cash transfers run the risk of only providing short-term relief with no concrete long-term outcomes as a result of not addressing underlying structural and systemic issues (such as the *θ* parameter, which prohibits project execution when too high).

## Discussion

The transition from extreme poverty to extreme inequality highlights the importance of addressing various interconnected challenges to achieve Sustainable Development Goals effectively. Here, we present a pathway that outlines steps toward achieving sustainable development while addressing both poverty and inequality.

### Inequality drives persistent poverty

In the Introduction, we made two observations. First, although extreme poverty has decreased in the last three decades, recently about 97 million more people are living on less than $1.90 a day due to the COVID-19 pandemic, increasing the global poverty rate from 7.8 to 9.1 percent. Globally, three to four years of progress toward ending extreme poverty are estimated to have been lost. Second, between-country inequality is estimated to be increasing for the first time in a generation. Emerging evidence even shows that within countries, inequality may also have worsened. The key question is: are we stuck between extreme poverty and extreme inequality? Why is progress so vulnerable to shocks?

An examination of the parameter space reveals that our model captures two extreme regimes: one characterized by poverty and the other prosperity. Both regimes have low inequality. An interesting third regime emerges where some agents are trapped in poverty while others can escape poverty and accumulate wealth. An analysis of the Gini coefficient of simulations in this regime shows that inequality is negatively correlated with economic development (see Fig. 5 in Appendix F)—a fact well established in empirical literature (Alfani and Schifano, 2021). High inequality inhibits social mobility perpetuated through increased segregation in social connections, high cost to access financial services, sub-optimal diversification and low returns to investment. Although our analysis primarily focuses on agents escaping poverty (a form of upward absolute mobility), a different approach to measuring social mobility could involve measuring rank dynamics, such as the “stickiness” of an agent’s or community’s position within the wealth distribution over time, as suggested by recent literature (Stojkoski, 2024). Our findings in “Asset-based interventions are context dependent,” where some interventions allow agents to escape poverty in the Some Rich regime, provide initial insights into factors promoting such upward movements. Future work could explicitly quantify these transition dynamics.

While there are clear pathways in the parameter space to move from the All Poor regime to All Rich via the Some Rich regime, our model indicates two key challenges. First, progress in poverty reduction is vulnerable to shocks due to the presence of critical transitions, wherein even a small change in the parameter space can lead to regime shifts. Many poverty reduction programs rely on sustained economic growth and stability of the parameter space. Economic shocks, such as recessions, currency devaluations, or commodity price fluctuations, can disrupt growth trajectories and undermine the gains made in poverty alleviation. Lack of savings, access to credit, or insurance can magnify the impact of such shocks, leading to deeper and more prolonged periods of poverty. In an interconnected world, shocks in one part of the world can quickly ripple through to other regions. We show that in a multi-dimensional parameter space, there are multiple pathways through which shocks can cause regime shifts in the economic landscape. Second, in unequal societies, powerful individuals or groups may engage in rent-seeking behaviour, using their influence to capture economic rents or extract resources from the rest of society without creating corresponding value. This can exacerbate inequality by diverting resources away from productive uses and concentrating wealth in the hands of a few. The intersection of inequality and political economy is a rich area of study that examines how economic policies, institutions, and power dynamics shape and perpetuate unequal distributions of resources, opportunities, and outcomes within societies. While we do not model such a feedback explicitly, the analysis of the homophily parameter in our model is an excellent proxy for the political economy and validates this finding. Finally, horizontal inequalities (see Fig. 7 in Appendix G) refer to disparities among distinct identity groups and communities, such as racial divides between blacks and whites, gender disparities between women and men, religious differences like Muslims and Hindus, or ethnic tensions like Hutus and Tutsis, among various other examples. These inequalities are inherently unjust and often endure over time. Our results indicate that horizontal inequality is significantly higher than vertical inequality. This demonstrates that an individual’s position in the distribution is mainly determined by the community (group) they belong. Deprived groups face formidable obstacles, including limited financial resources for investing in assets and education for their children, as well as restricted access to loans. Additionally, social networks typically remain segregated within these groups, resulting in fewer beneficial connections for individuals from disadvantaged backgrounds seeking access to quality education or employment opportunities (Roy et al., 2018a). The Sustainable Development Goals acknowledge the imperative of addressing inequalities not only at the individual level but also among different identity groups.

As discussed next, addressing these vulnerabilities requires a multi-dimensional approach that includes building resilience at individual, community, and institutional levels, implementing adaptive policies, and fostering sustainable development strategies that are less susceptible to external shocks.

### Pathways to poverty alleviation and social mobility

What types of poverty alleviation interventions are possible, and how do they depend on the different pathways of inequality and poverty found at different societal scales? In recent work (Lade et al., 2017), using a resilience-thinking lens, describe three different types of interventions that assume the existence of a barrier to escaping poverty.

The first type of interventions involves capital inputs to the poor state in order to “push it” over existing barriers, for example, cash transfers or increased agricultural inputs. Although effective in some cases, it is important to note that such interventions do not lead to a corresponding systemic change and therefore may not yield substantial long-term benefits. Our intervention experiments on artificial societies in the All Poor regime appear to replicate this particular phenomenon: mere capital injection did not alter the fundamental unfavourability of the investment landscape (e.g., high project cost or poor project returns) or harmful behavioural patterns (e.g., high consumption, low saving propensity) that defined the All Poor trap. While a cash transfer might enable an agent to overcome project costs temporarily, if this cost itself isn’t reduced or if project viability does not improve, the agent is likely to fall back into poverty after some time. Furthermore, as shown in our results on the impact of heterogeneity in the agent population (in the form of attention and utility parameters), additional factors such as access to information and education can be important in improving an individual’s chances of escaping poverty. Furthermore, money transfer programs can sometimes encounter moral hazard issues. Moral hazard refers to the risk that individuals or organisations might change their behaviour in response to the assurance of financial assistance, leading to unintended consequences.

The second type of intervention consists of lowering the barrier by changing the parameters that otherwise serve as key mechanisms in inducing and reinforcing poverty. Although reducing the risk aversion of individuals and communities such that they undertake riskier ventures is technically possible in our modelling framework, in practice, this may not be the case. Individuals may not be willing to accept higher levels of risk when already facing the obstacles of extreme poverty or the near prospect of them. By contrast, we find that improving financial inclusion and access to markets or facilitating the adoption of climate-smart farming practices could realistically help lower difficulties associated with escaping the poverty cycle at the individual and community levels. Further, we show that promoting diversity can also lead to lowering the barrier. Homophily can reinforce existing inequalities by perpetuating patterns of advantage and disadvantage within social networks. For example, individuals from privileged backgrounds may have greater access to social networks with valuable connections and opportunities, while those from marginalized or disadvantaged backgrounds may face barriers to entry or exclusion from such networks. As a result, inequalities in access to resources, opportunities, and social capital can become entrenched and perpetuated across generations. Overall, diversity and access to technology empower individuals, strengthen communities, and foster inclusive and sustainable development. By leveraging technology effectively, policymakers, businesses, and civil society organisations can advance efforts to reduce poverty and inequality and build a more equitable and prosperous future for all.

Lastly, the third type of interventions involves major systemic transformations that bring about fundamentally different poverty and inequality landscapes. An example of such an intervention would be to provide novel developmental pathways to communities while still adhering to key tenets of social and environmental justice (Lade et al., 2017). One of the key findings of our model is the emergence of sub-optimal diversification. Addressing sub-optimal diversification often involves systemic transformation and broader strategies aimed at innovations (e.g., innovative agricultural practices), improving access to education and skills training (e.g., new challenge-based learning), fostering entrepreneurship (e.g., microloans, savings groups), and building resilience to external shocks. These measures can help break the cycle of poverty and create more sustainable livelihoods. Although this type of intervention is arguably the most challenging to implement, it has the potential to yield the greatest benefit since it encourages a reconfiguration of the relationships between agents, their environment, and economic practices, which can all contribute to persistent poverty if not appropriately addressed. For example, negative consequences could quickly arise from asset input into a community with prevalent gender inequality: while such an intervention could appear successful in terms of aggregate asset ownership, gender inequalities may intensify if women’s corresponding access is not ensured in a culturally sensitive manner (Simon, 2019).

Overall, we find that the complex and multi-level nature of poverty traps makes it especially difficult to generalise findings across different contexts: a one-size-fits-all policy or intervention simply does not exist. In a recent paper, Radosavljevic et al. discuss the existence of fractal poverty traps wherein “multiple low-level equilibria exist on different levels at the same time and self-reinforce through cross-level feedbacks” (Radosavljevic et al., 2021). These cross-level dynamics are particularly crucial to understand because interventions at the household level, for instance, could have unforeseen and damaging consequences at the community level. This concern points to the importance of integrating information from multiple levels to better understand and quantify how multi-level interactions contribute to poverty trap mechanisms. The metric we employ to define and track progress across levels is also of critical importance. For instance, although shown to be a poor indicator of quality of life, the gross domestic product remains one of the most commonly used metrics to compare national economies. Integrating alternative indicators such as the Multidimensional Poverty Index (UNDP, 2023) or the Green Growth Index, for instance, could provide a more comprehensive overview of how countries, cities, and communities are performing in terms of poverty reduction and sustainable development targets. Moreover, only looking at the global Gini coefficient for income per capita disguises the glaring inequalities that we have argued still exist at regional and country levels. In fact, it is easy to show that a unimodal income distribution, such as the one we see today at the global level, may be constructed by summing many bimodal distributions exhibiting inequality (see Appendix F and Fig. 6).

## Supplementary information

Supplementary information

## Data Availability

This study does not use any real-world data; all results are generated from simulation experiments. The complete set of simulation outputs generated and analysed during the current study are available in the Figshare repository: https://figshare.com/s/23efbca44d7b28ac0340.The simulation model, along with all scripts necessary to reproduce the results and generate all figures presented in the paper, is available as open-source code at https://github.com/charlesaugdupont/poverty-trap.

## Code availability

This study does not use any real-world data; all results are generated from simulation experiments. The complete set of simulation outputs generated and analysed during the current study are available in the Figshare repository: https://figshare.com/s/23efbca44d7b28ac0340. The simulation model, along with all scripts necessary to reproduce the results and generate all figures presented in the paper, is available as open-source code at https://github.com/charlesaugdupont/poverty-trap.
