# Supplementary material for: Emergent poverty traps at multiple levels impede social mobility
Source: Humanit Soc Sci Commun. 2025 Nov 19;12(1):1777. doi: 10.1057/s41599-025-06089-9 (PMC12629999; doi:10.1057/s41599-025-06089-9)
Supplement: Supplementary file 1 — Supplementary information [file 41599_2025_6089_MOESM1_ESM.pdf]

Supplementary Information: Emergent poverty traps at multiple levels impede social mobility

Appendix A: Poverty Traps and Economic Modelling

Barrett et al. (2013), argue that poverty traps can be generated by a variety of structural mechanisms. They could be found at the individual or household level, while some operate at the community, regional, or national scales. Some are single equilibrium mechanisms while others are multiple equilibrium mechanisms (Barrett and Carter, 2013). A single equilibrium poverty trap occurs when a person or community remains poor forever, such that there is no other stable state. Whereas multiple equilibrium poverty traps exist when an individual or community fluctuates between the states of being poor or non-poor, or if there exist multiple stable states (Barrett and Carter, 2013). There are one-dimensional or multi-dimensional poverty trap models depending on the number of dimensions of poverty that are being studied. Barrett et al., in their multi-dimensional poverty trap model, incorporate human capital which they define as 'the innate ability that includes the physical stature, cognitive development, and the level of education with which a person enter adulthood' along with financial capital. They applied the concepts of 'regime shift' in two contexts: 'social protection' – individuals adjusting their behaviour in response to poverty alleviation methods in presence of a shock – and 'social relief' – individuals not changing their behaviour in response to the policies implemented to support individuals in the presence of a shock. Barrett et al. give an example of implementing 'crossing barrier' behaviour of regime shift in their study titled 'Poverty Traps and Social Protection'. The 'Need-Based Assistance' where each individual is given financial support to attain a higher income stage was implemented as a 'social relief' policy and 'safety nets' – used to prevent agents from falling below a threshold and 'cargo net' – money transfers made to lift people above threshold, were implemented as 'social protection' policy (Barrett et al., 2008; Ikegami et al., 2017). Their one of a kind model has been an inspiration for the model developed during the course of this thesis project. Azariadis (Azariadis and Stachurski, 2005) et al. reviews mechanisms that create self-reinforcing traps preventing technological adoption. Key factors include: Incentive structure that discourage modern sector investments and skill acquisition, credit market imperfections trapping the poor in low-return activities, rent-seeking behaviours and social conventions (Azariadis and Stachurski, 2005). These mechanisms suggest development is not automatic, as small initial differences can amplify and persist, contrasting sharply with neoclassical growth models.

Here, we present the different types of multi-dimensional multiple equilibrium poverty trap models that exist in the literature. Among the many existing models, we can use a broad categorization of System Dynamics Models and Agent-Based Models. Steven et al., developed a multi-dimensional, process-based, dynamical, multiple equilibrium poverty trap model in the premise of rural agricultural communities (Lade et al., 2017). They considered Natural Capital, Physical Capital, and Cultural Capital as the dimensions for their multi-dimensional multiple equilibrium poverty trap model. The choice of the capitals is justified owing to the rural agricultural scenario that formed the subject of study. The models developed by Steven et al., are unique in that they apply the resilience thinking concepts of regime shift and transformation to develop poverty alleviation methods on their multi-dimensional multiple equilibrium poverty trap model (Lade et al., 2017). The model formulated by Steven et al. studied poverty trap formation at a rural community level. They also mention how an exogenous shock like flooding affects the already weak natural capital in the case of the Intensification Trap Model (Lade et al., 2017). This in turn led to the adoption of methods that make the natural capital less susceptible to floods at the community level (Lade

et al., 2017). Brinkmann and his colleagues developed an agent-based model to understand the dynamics of social-ecological traps in the South Western Madagascar where the farmers are negatively affected by the unsustainable exploitation of natural resources. The area witnessed a dramatic increase in the land use pressure on resources over the past years. They illustrated a model-driven scenario and devised methods to help the farmers escape this trap (Brinkmann et al., 2021).

Appendix B: Global Sensitivity Analysis

In this appendix, we investigate what parameters our model is most sensitive to in order to better understand underlying model mechanics and processes. In order to accomplish this, we perform global sensitivity analysis using recent work by Bazyleva et al. (Bazyleva et al., 2023), where the authors employ Grassmannian diffusion maps and polynomial chaos expansion to estimate sensitivity indices. As demonstrated by the authors, this approach is particularly powerful for analysing agent-based models since such models exhibit rich temporal dynamics over multiple scales and are intrinsically stochastic.

Figure 1 shows the (three-dimensional) diffusion coordinates obtained for one of twenty stochastic repetitions of our experiment. In the top and bottom rows, we find the coordinates produced at the micro, and macro levels respectively. Furthermore, the chosen manifold dimension p for each level was 91 and 3 respectively.

Figure 2 displays the first- and total-order sensitivity indices of all five parameters for the top three diffusion coordinates at the micro and macro levels. It is important to point out that, in general, the first $\boldsymbol{\theta}$ corresponds to short-scale temporal dynamics, the second $\boldsymbol{\theta}$ corresponds to longer time scales, and, finally, the third $\boldsymbol{\theta}$ mostly captures additional noise. At both the micro and macro levels, θ appears to have the highest single and total sensitivity indices along the first diffusion coordinate. This makes intuitive sense, as θ modulates the investment required for a project to be carried out by a community, and therefore directly impacts the system's evolution at every time step. First-order sensitivity indices are near zero for all parameters along the second and third diffusion coordinates. However, all parameters have similar *total* order sensitivity indices at both micro and macro levels for the second and third diffusion coordinates. In particular, we can observe that the homophily parameter α has the highest total-order sensitivity indices along these coordinates, implying that it plays an important role in longer time scales. This is also a sensible outcome, since homophily impacts network and community structure, which play a longer-term role in how wealth gradually becomes distributed throughout the network. Lastly, the large differences found between first and total order sensitivity indices point to significant interactions between the parameters.

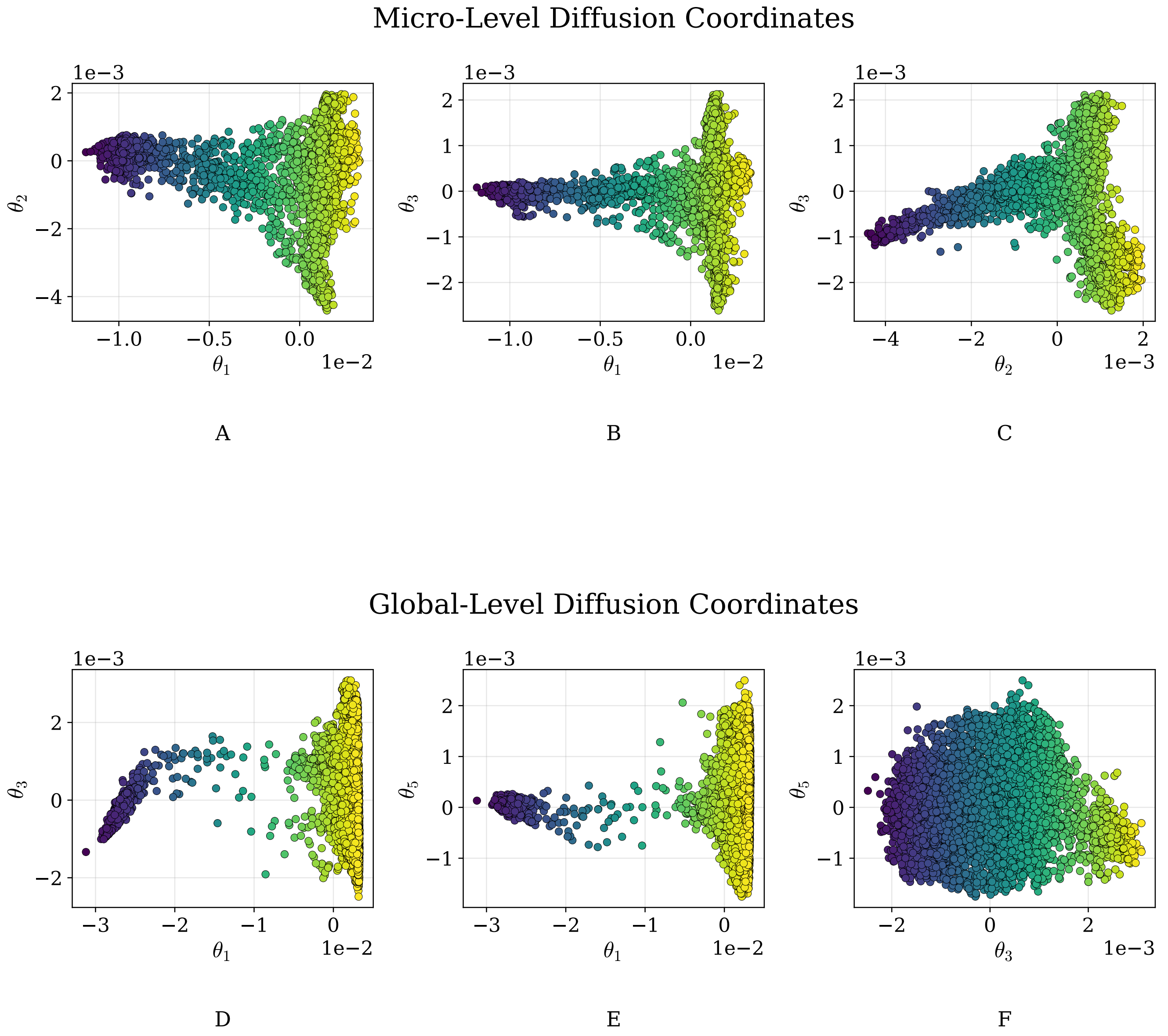

Figure 1. Diffusion coordinates obtained from applying Grassmannian diffusion maps at the micro and macro levels, similar to work by Bazyleva et al. (Bazyleva et al., 2023) These results are for just one of 20 stochastic repetitions of our experiment. **A:** Projection of micro-level diffusion coordinates along θ_1 and θ_2. **b:** Projection of micro-level diffusion coordinates along θ_1 and θ_3. **C:** Projection of micro-level diffusion coordinates along θ_2 and θ_3. **D:** Projection of macro-level diffusion coordinates along θ_1 and θ_3. **E:** Projection of macro-level diffusion coordinates along θ_1 and θ_5. **F:** Projection of macro-level diffusion coordinates along θ_3 and θ_5.

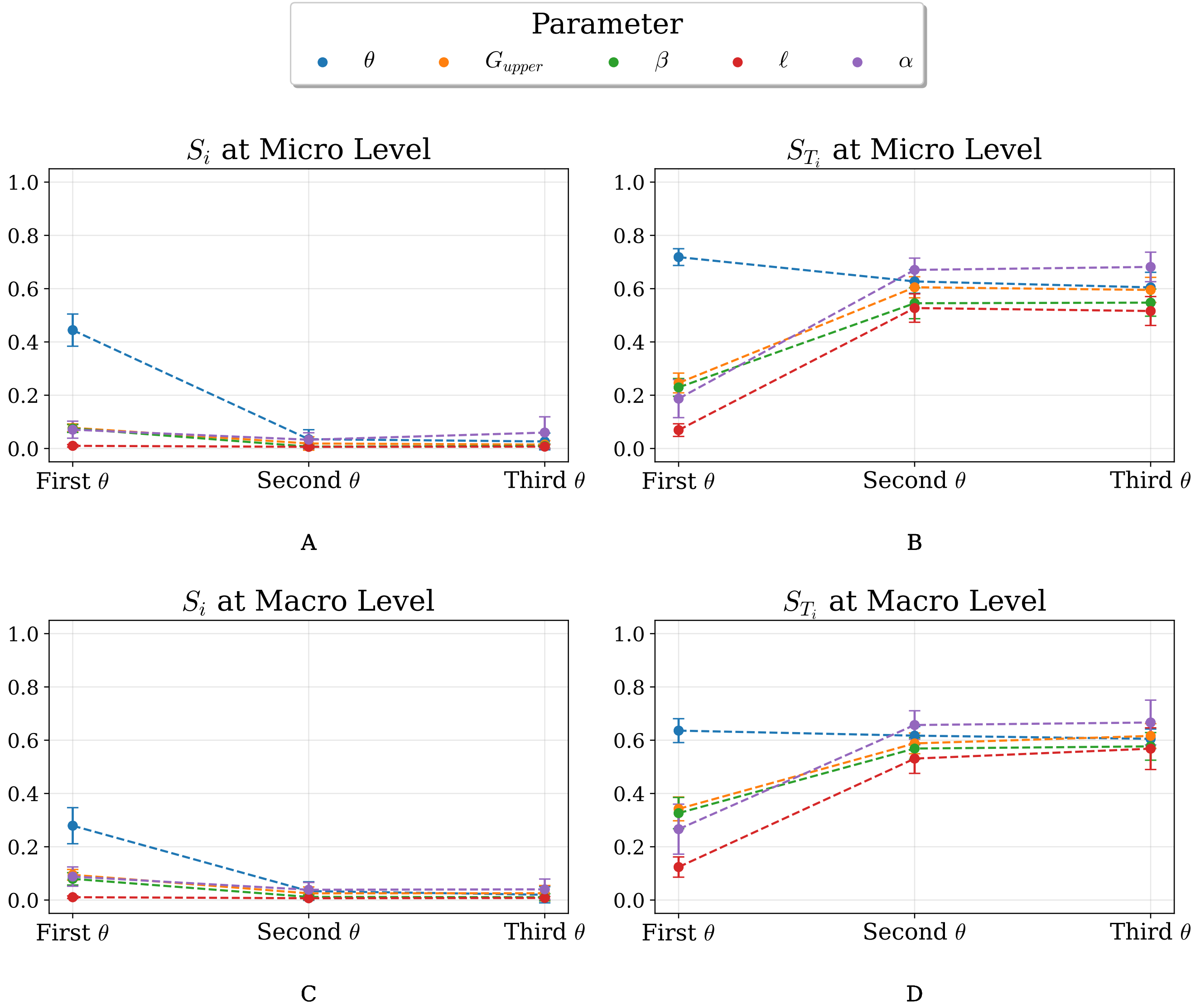

Figure 2. First and total order sensitivity indices of key model parameters – θ (project investment threshold), G_{upper} (project gain upper bound), β (saving propensity), ℓ (project loss probability), and α (homophily in network formation) – at micro (agent) and macro (population) levels. The indices are computed using Grassmannian diffusion maps and polynomial chaos expansion applied to the ensemble of all 7168 parameter combinations, each with 20 stochastic repetitions. The maximum degree of PCE polynomials is $s = 15$. The model exhibits particular sensitivity to θ (project cost) and α (homophily). Total order indices indicate a high degree of interaction between parameters. **A**: First order sensitivity indices of parameters at the micro level. **B**: Total order sensitivity indices of parameters at the micro level. **C**: First order sensitivity indices of parameters at the macro level. **D**: Total order sensitivity indices of parameters at the macro level.

Appendix C: Key Distributions for Generated Social Distance Attachment Networks

In this appendix, we analyse some important distributions for the Social Distance Attachment networks that were generated for our experiments. Looking at Figure 3 (left), we can see that communities typically have less than 250 agents, although we found an instance of a community containing 1215 agents (almost the entire population). Additionally, this distribution appears to follow a power law, which is typical of the social distance attachment mechanism. The central plot reveals that most communities are connected to only a few other communities, although some exhibit very high connectedness. Lastly, the rightmost plot shows that the number of communities in any given experiment is usually rather small, with 26 being the most common amount. Note that the community size and degree distributions follow a roughly power-law like decay.

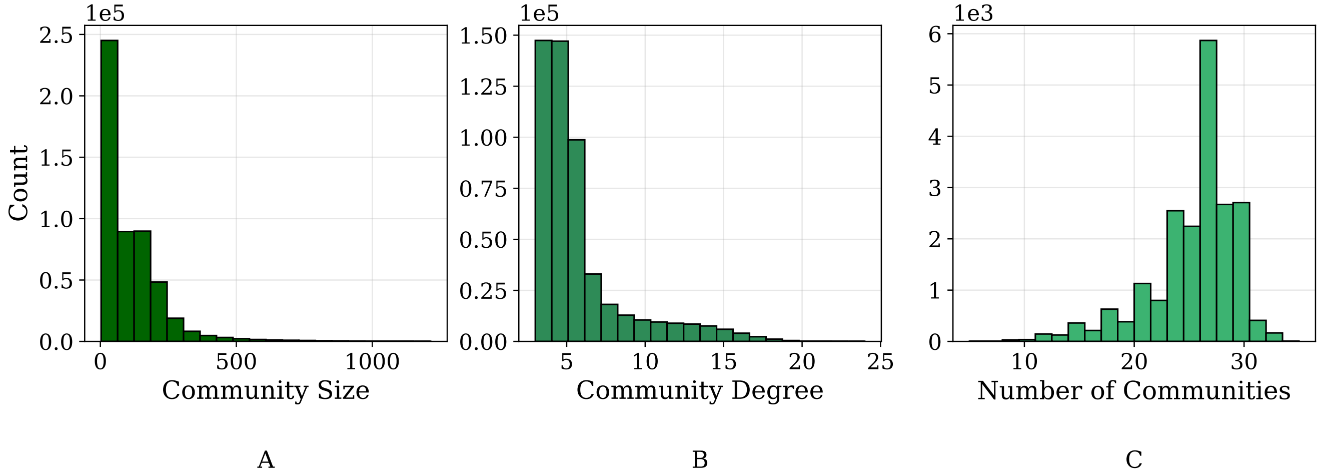

Figure 3. Key statistical distributions for the generated Social Distance Attachment (SDA) networks. Data are pooled from all SDA graphs constructed across all 7168 parameter combinations. **A:** Distribution of community sizes. **B:** Distribution of community degree (number of communities that a given community is connected to). **C:** Distribution of the number of communities.

Appendix D: Distribution of Average Project Returns by Regime

In this appendix we present the average project returns generated across all parameter combinations and repetitions of our experiment. This analysis occurs at the meso (community) level since projects are assigned to each community and Figure 4 displays the results. In the All Poor regime, average project returns are all below 2.5, with a significant proportion being zero, which implies that projects tend to fail frequently and rarely yield gains. The Some Rich regime exhibits the largest spread of average project returns out of all three regimes. Many projects still tend to fail, but many also succeed on average and yield high gains. Interestingly, simulations in the All Rich regime do not exhibit average project returns as high as the Some Rich regime, although this could in part be due to the low proportion of simulations observed in this regime (< 0.1%). Lastly, in this regime, project returns are always greater than 1 on average and therefore always favourable to investors.

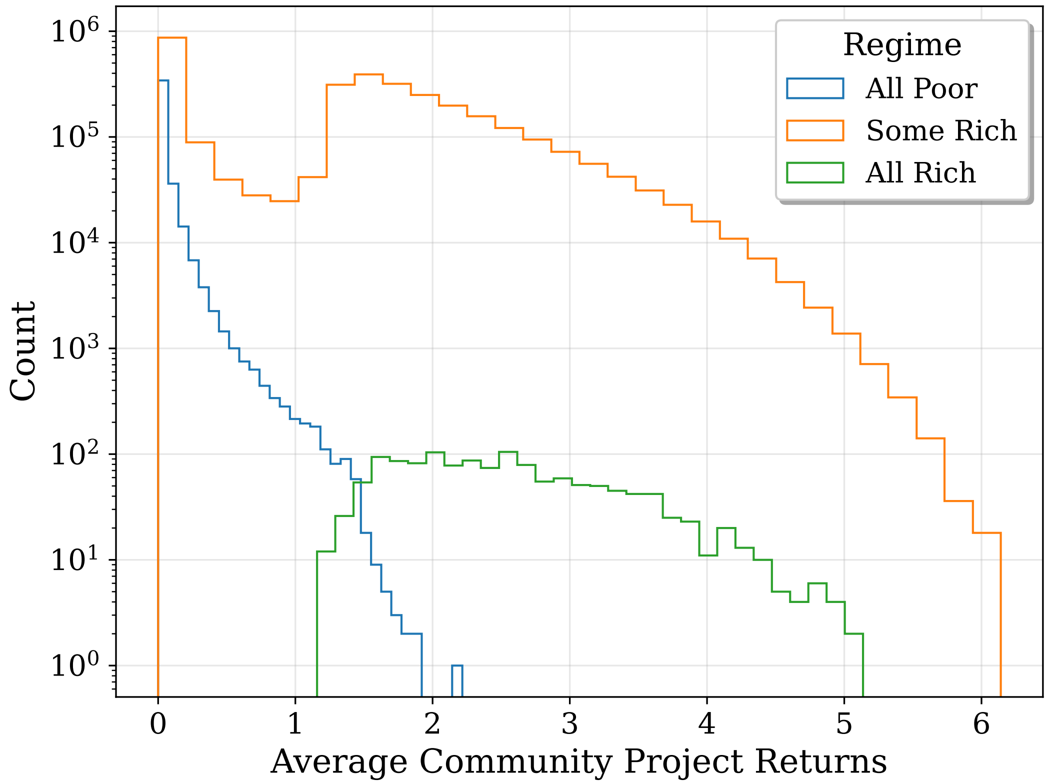

Figure 4. Distribution of average project returns at the community (meso) level. Data are pooled from all projects across all simulation runs (7168 parameter combinations $\times$ 20 repetitions), with results categorized by the agent-level regime (All Poor, Some Rich, All Rich) observed in that run. All Rich regime projects appear to always succeed (no returns below 1), whereas the Some Rich and All Poor regimes exhibit many instances of project failure. Furthermore, while project returns decay rapidly for the All Poor regime, the Some Rich returns give rise to a more bimodal distribution with very high returns in the case of some projects.

Appendix E: Relationship Between Final Total Population Wealth and Gini Index for the Some Rich Regime

In this appendix we highlight the relationship between final wealth (aggregated over the entire population) and the Gini index for the Some Rich regime, which exhibits Gini indices across the entire spectrum of values from 0 to 1 (as seen in **??**). Figure 5 demonstrates a clear decrease in Gini index as the total population wealth increases. If not for the logarithmic scaling of the x-axis, used here for improved visualization of details, we would see a very strong negative linear correlation (Pearson $r = -0.998$). This result makes sense intuitively in the Some Rich regime – since some agents are bound to have accumulated wealth beyond their starting point, if the total final wealth is very low, then we expect a high degree of inequality, as opposed to when the total wealth is very high (meaning that almost all agents are rich).

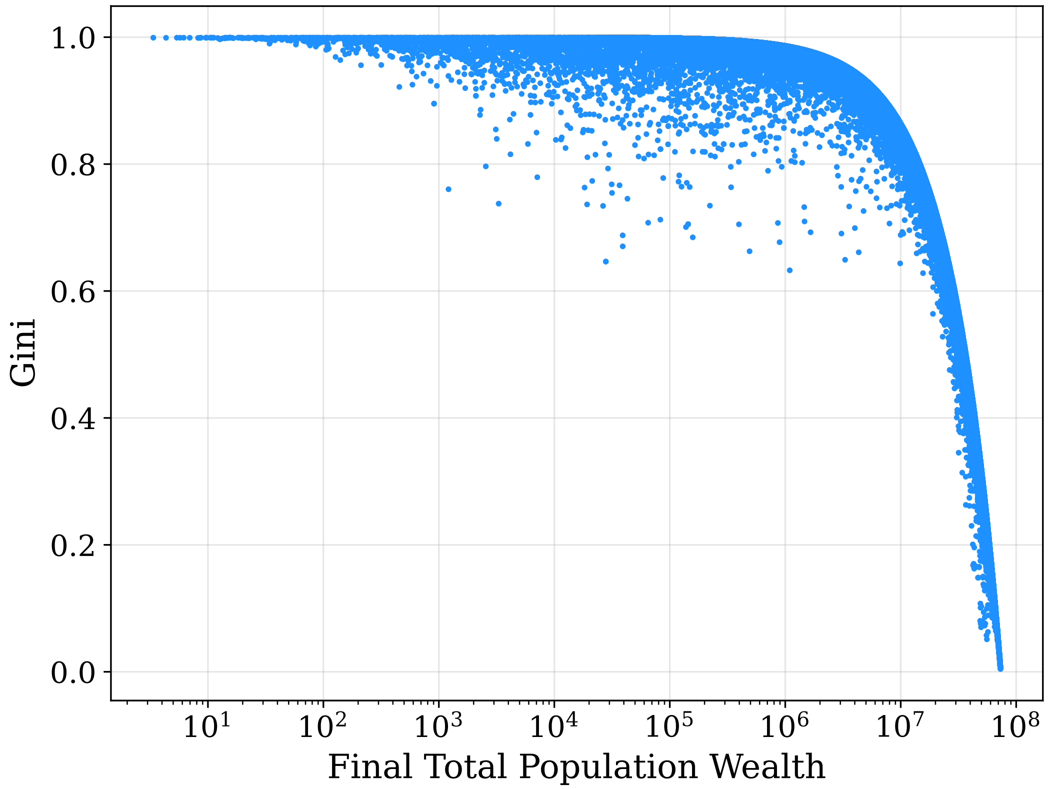

Figure 5. Relationship between final total population wealth and Gini index for all simulation runs that resulted in the Some Rich regime. Each point represents the outcome of one simulation run (from the 7168 parameter sets x 20 repetitions, filtered for the Some Rich regime). As the final population wealth increases increases, inequality – and therefore the Gini index – decreases linearly (not visible here due to logarithmic scaling of the x-axis).

Appendix F: Summation of Bimodal Distributions

This appendix serves as a simple demonstration of how multiple bimodal distributions may be added to give rise to a unimodal distribution. A bimodal distribution is generated by randomly drawing $N = 1000$ samples each from two normal distributions, $\mathscr{N}_1$ and $\mathscr{N}_2$. The mean and standard deviation of these distributions are randomly drawn from uniform distributions $U_\mu(500, 800)$ and $U_\sigma(10, 50)$ respectively. The left plot of Figure 6 shows such a bimodal distribution. By generating and summing additional bimodal distributions, we are able to obtain a progressively tighter (middle plot) and eventually seemingly unimodal distribution (right plot). Although a toy example, this outcome is reminiscent of the way in which the global wealth and income distributions appear unimodal, thereby obscuring the high levels of within- and between-country inequality that persist even today.

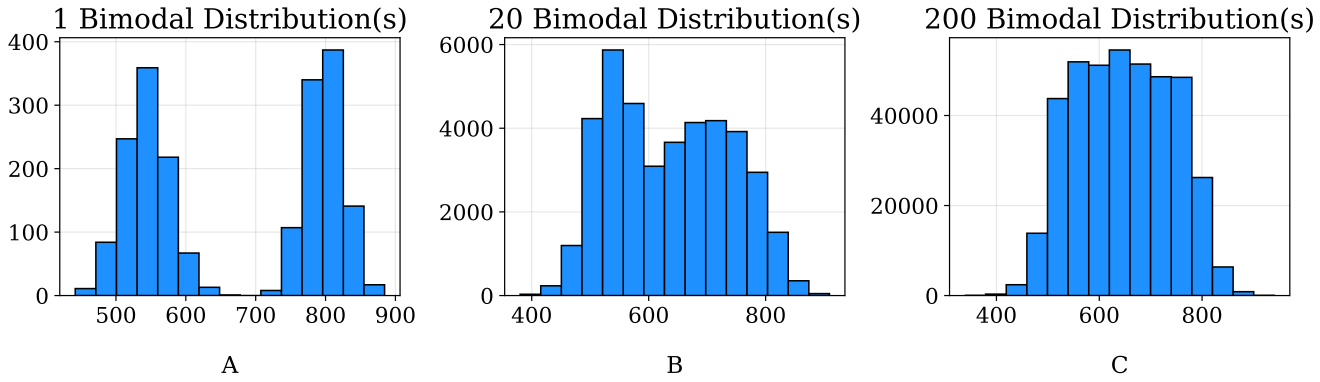

Figure 6. The sum of many bimodal distributions can appear to converge towards a unimodal one. **A:** A single instance of a bimodal distribution. **B:** Sum of 20 bimodal distributions. **C:** Sum of 200 bimodal distributions.

Appendix G: Horizontal Inequality

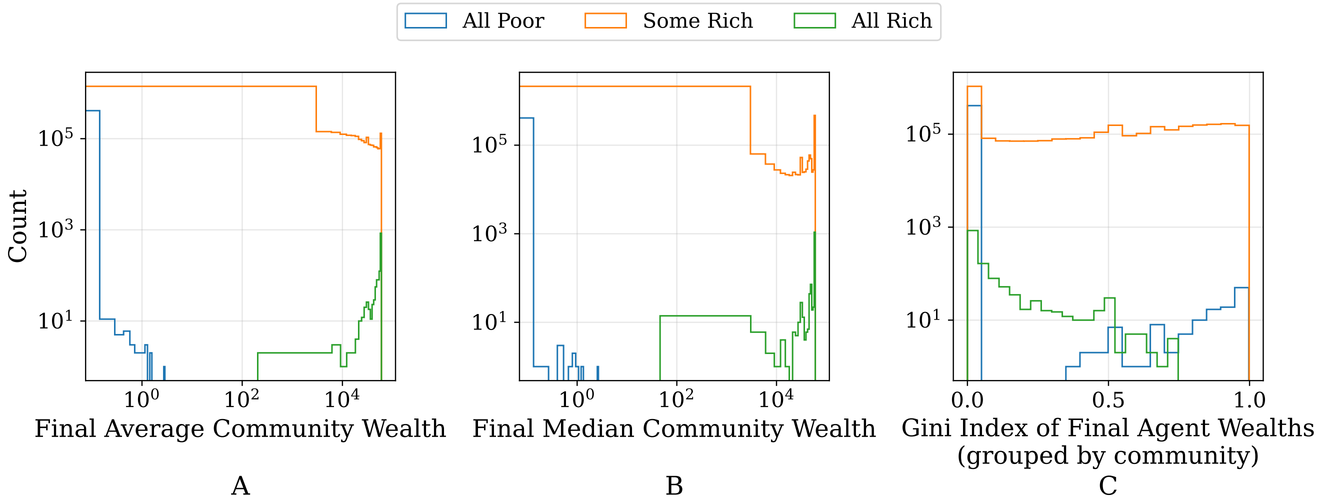

Figure 7. Wealth and inequality distributions at the community level, categorized by community-level regime definitions (All Poor, Some Rich, All Rich). Data are pooled from all communities across all simulation runs (7168 parameter combinations $\times$ 20 repetitions). In the All Poor regime, each community's final total wealth is less than its total initial wealth. Meanwhile, in the Some Rich regime some communities have final total wealth greater than total initial wealth and all communities have higher final total wealth in the All Rich regime. The leftmost and central plots respectively show final average and median community wealth in these three regimes. The rightmost plot shows the distribution of inequality between communities (horizontal inequality) – note that although the All Poor regime exhibits instances of high Gini indices, this is primarily due to rounding inaccuracies (all agents have wealth close to zero, but some may have a small positive wealth of e.g. 10^{-5}.

Appendix H: Empirical and Simulated Complementary Cumulative Distribution Function (CCDF)

This appendix aims to compare our simulated steady-state wealth distributions under the Some Rich regime with empirical wealth distribution data from the UK (Office for National Statistics, 2016). Since the model simulation and empirical wealth data have different scales, we normalize values to $[0,1]$ using $1-e^{-kx}$, with $k = 0.0008$. Figure 8 displays this comparison for 50 of our simulations that have the smallest Kolmogorov-Smirnov distance (when comparing CCDFs) with respect to the empirical distribution, and we observe that there is overall good alignment. For reference, the simulation that yielded the CCDF closest to the empirical distribution had parameters: $\theta = 0.66$ (determines project cost) $G_{\text{upper}} = 2.85$ (project gains), $\beta = 0.78$ (saving propensity), $\ell = 0.34$ (loss probability), $\alpha = 3.65$ (homophily). This parameter combination corresponds to relatively high project costs, low homophily (more initial connections between agents with dissimilar wealth levels), and high saving propensity.

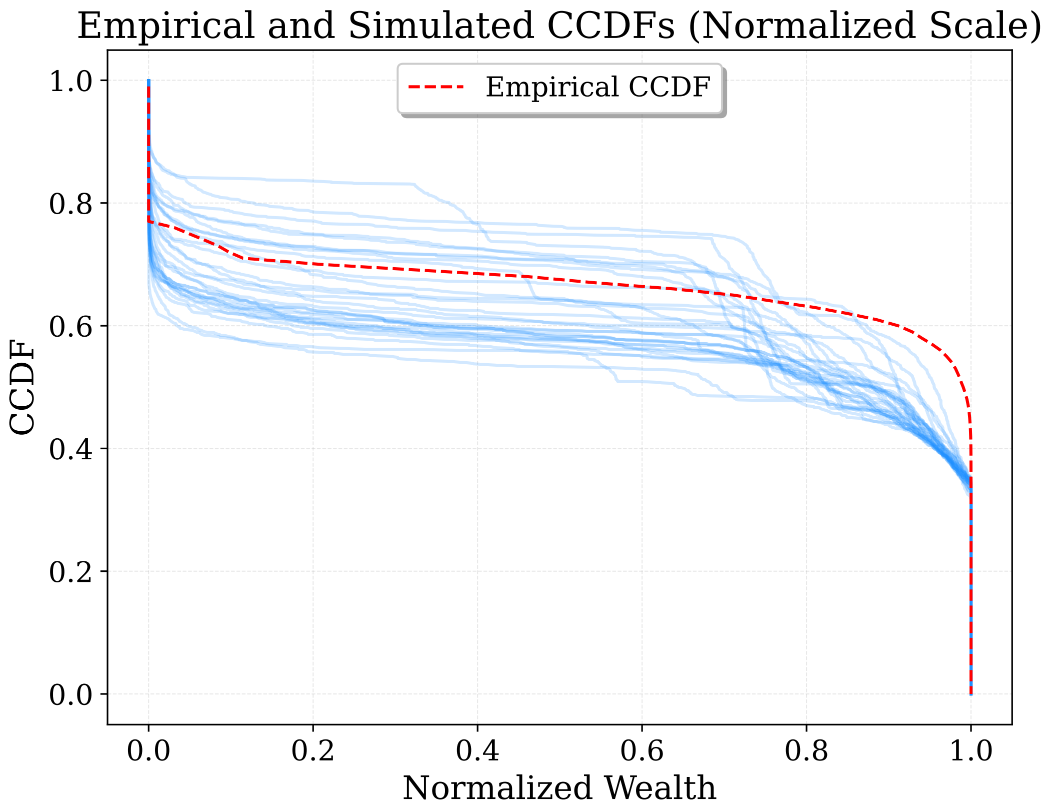

Figure 8. Comparison of empirical and simulated Complementary Cumulative Distribution Functions (CCDFs) for wealth on a normalized scale. The dashed red line represents the empirical CCDF derived from UK wealth distribution data. The multiple light blue lines depict the CCDFs from a sample of simulation runs categorized under the Some Rich regime of our agent-based model, where wealth is normalized. The plot shows a good overall agreement in the shape of the distributions, indicating that the model's Some Rich outputs are comparable to observed empirical wealth inequality patterns.

References

Costas Azariadis and John Stachurski. Poverty traps. *Handbook of economic growth*, 1:295–384, 2005. doi: 10.1016/S1574-0684(05)01005-1.

Christopher B Barrett and Michael R Carter. The economics of poverty traps and persistent poverty: Empirical and policy implications. *The Journal of Development Studies*, 49(7):976–990, 2013. doi: 10.1080/00220388.2013.785527.

Christopher B Barrett, Michael R Carter, and Munenobu Ikegami. Poverty traps and social protection. *Available at SSRN 1141881*, 2008.

Valentina Bazyleva, Victoria M. Garibay, and Debraj Roy. Global sensitivity analysis using polynomial chaos expansion on the grassmann manifold. In Jiří Mikyška, Clélia de Mulatier, Maciej Paszynski, Valeria V. Krzhizhanovskaya, Jack J. Dongarra, and Peter M.A. Sloot, editors, *Computational Science – ICCS 2023*, pages 583–597, Cham, 2023. Springer Nature Switzerland. ISBN 978-3-031-36030-5. doi: 10.1007/978-3-031-36030-5_46.

Katja Brinkmann, Daniel Kübler, Stefan Liehr, and Andreas Buerkert. Agent-based modelling of the social-ecological nature of poverty traps in southwestern madagascar. *Agricultural Systems*, 190: 103125, 2021. ISSN 0308-521X. doi: https://doi.org/10.1016/j.agsy.2021.103125. URL https://www.sciencedirect.com/science/article/pii/S0308521X21000780.

Munenobu Ikegami, Michael R Carter, Christopher B Barrett, and Sarah Janzen. Poverty traps and the social protection paradox. In *The economics of poverty traps*, pages 223–256. University of Chicago Press, 2017.

Steven J. Lade, L. Jamila Haider, Gustav Engström, and Maja Schlüter. Resilience offers escape from trapped thinking on poverty alleviation. *Science Advances*, 3(5), 2017. doi: 10.1126/sciadv.1603043.

Office for National Statistics. Total wealth: Wealth in great britain - july 2006 to june 2016, 2016. URL https://www.ons.gov.uk/peoplepopulationandcommunity/personalandhouseholdfinances/incomeandwealth/datasets/totalwealthwealthingreatbritain/july2006tojune2016.
